# The Impact of Economic Recessions on Depression, Anxiety, and Trauma-Related Disorders and Illness Outcomes—A Scoping Review

**DOI:** 10.3390/bs11090119

**Published:** 2021-08-31

**Authors:** Olivia Guerra, Ejemai Eboreime

**Affiliations:** Department of Psychiatry, University of Alberta, Edmonton, AB T6G 2R3, Canada; eboreime@ualberta.ca

**Keywords:** economic recession, mental health, depression, anxiety, trauma, suicide, mortality, scoping review

## Abstract

In the wake of a global economic recession secondary to the COVID-19 pandemic, this scoping review seeks to summarize the current quantitative research on the impact of economic recessions on depression, anxiety, traumatic disorders, self-harm, and suicide. Seven research databases (PsycINFO, MEDLINE, Embase, Web of Science: Core Collection, National Library of Medicine PubMed, PubMed Central, and Google Scholar) were searched for keywords returning 3412 preliminary results published since 2008 in Organisation for Economic Coordination and Development (OECD)nations. These were screened by both authors for inclusion/exclusion criteria resulting in 127 included articles. Articles included were quantitative studies in OECD countries assessing select mental disorders (depression, anxiety, and trauma-/stress-related disorders) and illness outcomes (self-harm and suicide) during periods of economic recession. Articles were limited to publication from 2008 to 2020, available online in English, and utilizing outcome measures specific to the disorders and outcomes specified above. A significant relationship was found between periods of economic recession and increased depressive symptoms, self-harming behaviour, and suicide during and following periods of recession. Results suggest that existing models for mental health support and strategies for suicide prevention may be less effective than they are in non-recession times. It may be prudent to focus public education and medical treatments on raising awareness and access to supports for populations at higher risk, including those vulnerable to the impacts of job or income loss due to low socioeconomic status preceding the recession or high levels of financial strain, those supporting others financially, approaching retirement, and those in countries with limited social safety nets. Policy makers should be aware of the potential protective nature of unemployment safeguards and labour program investment in mitigating these negative impacts. Limited or inconclusive data were found on the relationship with traumatic disorders and symptoms of anxiety. In addition, research has focused primarily on the working-age adult population with limited data available on children, adolescents, and older adults, leaving room for further research in these areas.

## 1. Introduction

Since the SARS-CoV-2 (COVID-19) pandemic was declared by the WHO on 11 March 2020, world economies have been hit by numerous unprecedented market closures, supply chain, trade, and finance interruptions leading to a global economic recession. The World Bank reported in June 2020 that the global economy would shrink by 5.2% in 2020—the deepest recession since World War II—and that economic activity among advanced economies was anticipated to shrink 7% [1]. Per capita incomes were expected to decline by 3.6%, tipping millions of people into extreme poverty with the most severe impacts in countries where the pandemic has been the most severe and there is heavy reliance on global trade, tourism, commodity exports, and external financing [1]. The Global Economic Prospects for 2020 warned of a lost decade, or more, of per-capita income gains and concern that cumulative factors, including massive public and private debt and a breakdown in education, will lead to a prolonged deterioration in economic prospects [2]. The 2021 Global Economic Prospects report predicts an expansion of 5.6% in 2021, the fastest post-recession pace in 80 years; however, global output will remain about 2% lower than pre-pandemic projections [3]. In this reality, the international community and governments around the world are looking to reboot their economies and put the recession of the COVID-19 pandemic behind us. Unfortunately, with history as our teacher, the repercussions of economic recessions are numerous, and the societal impacts are pervasive. There have been discussions throughout the pandemic about the impact of public health restrictions and the traumas of the pandemic itself on the mental health of our society; however, limited attention has been paid to the impacts of economic recessions on mental health.

Disaster mental health research has shown, over decades of research from the 1940s to the present, that following both natural and human-made disasters, specific psychological problems have been seen to occur, such as depression, anxiety, and trauma-related disorders [4,5,6]. Outcomes measured in the literature range from the presentation of increased symptoms to diagnoses of a psychiatric disorder, such as Major Depressive Disorder, Post Traumatic Stress Disorder (PTSD), or one of several anxiety disorders, as defined by the Diagnostic and Statistical Manual of Mental Disorders (DSM) [4]. Per the DSM5, symptoms of Major Depressive Disorder (MDD) include persistently depressed mood, diminished interest or pleasure in activities, change in appetite or weight, changes in sleep, psychomotor agitation or retardation, fatigue, feelings of worthlessness or guilt, impaired concentration, and recurrent thoughts of death or suicide [7]. PTSD includes exposure to actual or threatened death, serious injury, or sexual violence leading to persistent symptoms of intrusion, avoidance, negative cognitions and mood, hyperarousal, and/or altered reactivity [7]. Anxiety disorders, for example Generalized Anxiety Disorder (GAD), include a state of persistent and excessive anxiety or worry that is difficult to control, associated with physical symptoms (e.g., restlessness, fatigue, muscle tension, and insomnia) and changes in cognition and mood (e.g., impaired concentration and irritability) that cause significant distress or dysfunction [7].

While economic recession may not fit the common description of a disaster that is natural, such as earthquakes, forest fires, or floods, or human-made, such as war, terrorism, or train derailment, it certainly shares many of the consequences of such disasters, including financial loss, resource loss, housing issues or displacement, and stress [4]. Therefore, in this scoping review, we seek to identify and summarize the current evidence of the impact of economic recessions on the rates and characteristics of people who experience depression, anxiety, trauma-related disorders, as well as mental health outcomes related to these disorders (self-harm, suicidal ideation (SI), and suicide), and proxy measurements of this spectrum of depressive, anxious, or trauma-related symptoms and disorders, such as psychotropic drug use and the use of community or hospital-based mental health services, in developed nations. The intent is to explore the breadth of literature available and collate the evidence to further inform policy planning for prevention and protection of population mental health. In this study, we ask how recent economic recessions have impacted the mental health and mortality of the general population in democratic, free-market, first-world nations to increase awareness of at-risk populations during this current era of the global pandemic and recessions and inform efforts to improve surveillance and detection of these mental disorders and illness outcomes during current global recessions.

## 2. Materials and Methods

We performed a comprehensive search of PsycINFO, MEDLINE, Embase, Web of Science: Core Collection, National Library of Medicine PubMed, PubMed Central, and Google Scholar between 28 November and 28 December 2020 to identify relevant articles [8]. These sources of evidence were selected based on Bramer et al.’s 2017 study on optimal search strategies for comprehensive and efficient coverage of available literature when conducting systematic reviews [8]. The following search terms were used: “economic recession” AND ((“depressive disorder” OR “depression” OR “major depressive disorder” OR “major depressive episode” OR MDD) OR (“generalized anxiety disorder” OR GAD OR “anxiety”) OR (“post-traumatic stress disorder” OR “ptsd” OR “post traumatic stress disorder”) OR “mental health” OR “mental illness”).

All citations were imported into Proquest by the Refworks citation manager, which was used to sort and store article references. Abstracts were reviewed for each article and the reference list of excluded articles were reviewed for additional relevant literature. Inclusion criteria for articles were quantitative studies of all ages conducted in Organisation for Economic Coordination and Development (OECD) countries assessing mental disorders (excluding those exclusively assessing substance use) during periods of economic recession. Articles were limited to publication dates from 1 January 2008 up to the date that the search was completed (28 November–28 December 2020) to capture the most recent data, including that from the last major global economic recession in 2008.

Exclusion criteria included articles not relevant to the research question, including those using non-specific measures of mental distress or stress, or that were treatment or solution focused. Further excluded were articles that were opinion, editorial, commentary, or non-peer reviewed pieces, that used qualitative methodologies, were conducted in non-OECD countries, published pre-2008, or not available in English or online as these do not address the research question or would not be interpretable by the reviewers.

The authors independently screened the titles and abstracts of all studies identified through the search (primary screening). This was followed by secondary screening during which the full text of all studies that met the inclusion criteria in the primary screening were read and their eligibility to be included in the final review assessed.

Included articles were sorted by a hierarchy of evidence and publication date before study information and results were then collated and summarized by the primary author in a Microsoft Excel spreadsheet using study population, design, main outcomes measured, and significant findings related to the research question. These data points and methods were selected to most efficiently summarize the diverse foci and large volume of results included within these search parameters.

No assumptions or simplifications for data variables of search results collated were identified. A critical appraisal of the literature and sources of evidence were not completed during this review, as the aim of a scoping review is to explore the breadth of information available, rather than to assess the relative strength of findings or recommendations.

## 3. Results

**Figure 1:**
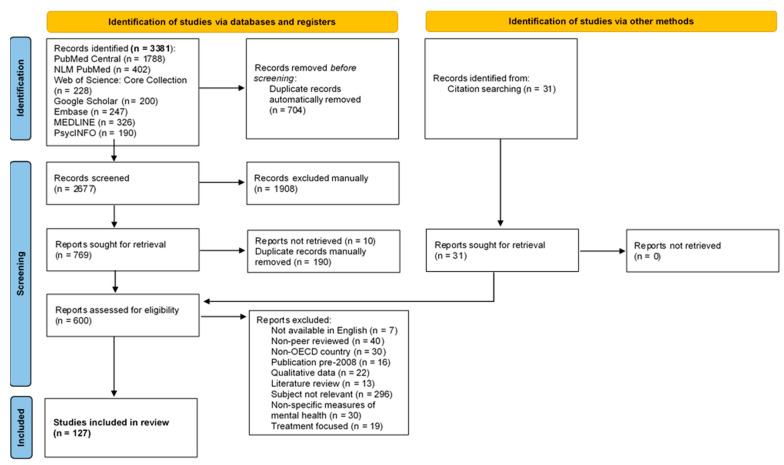
PRISMA literature search summary [9].

Using the above search parameters, a total of 3381 articles were initially included and imported into Proquest by Refworks citation manager. A total of 31 novel articles were identified by reference list review and imported into the citation manager for a total of 3412 articles reviewed by abstract. See Figure 1 for full details of the literature search [9], a supplementary literature search flowchart may also be found in the Supplementary Figure S1: Literature search flowchart. After the final body of 610 articles were screened for exclusion by two researchers, 127 articles were included in this systematic review. 

**Figure 2:**
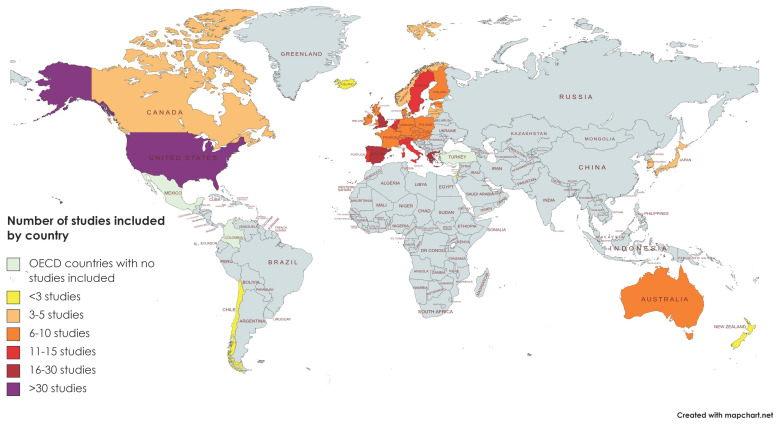
Geographical distribution of included studies.

Summary tables of these articles can be found by study topic in Table 1, Table 2, Table 3 and Table 4. An extended summary of included articles, can be found in Supplementary Table S1. A breakdown of study geography can be seen in Figure 2. Note that some studies include more than one geographical area and are counted in multiple categories.

Of the 127 articles included, there were 11 (9%) prospective cohort studies, 3 retrospective cohort studies, 1 case-control study, 84 (66%) time-trend analyses, and 28 (22%) cross-sectional analyses.

**Figure 3:**
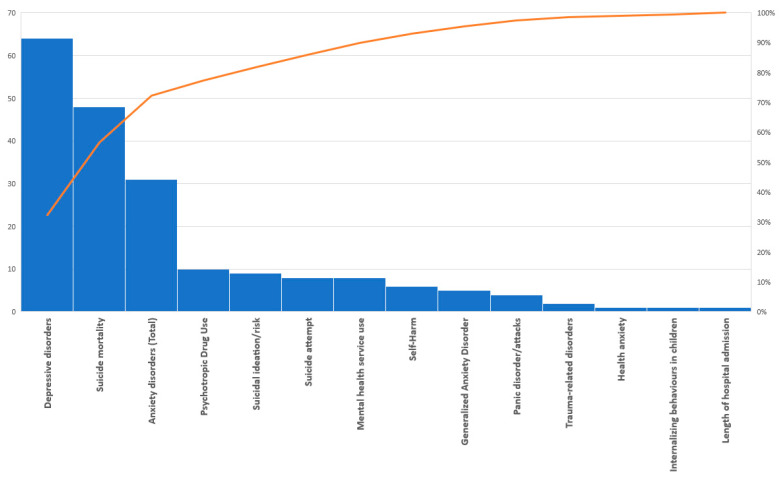
Pareto chart showing the distribution of included papers by outcomes studied.

As shown in the pareto analysis (Figure 3), depressive disorders, suicide mortality, and anxiety disorders were the by far the most reported outcomes, accounting for about 70% of the articles included. More specifically, 65 (50%) studies included outcomes on depressive symptoms, 48 (38%) on suicide mortality, and 21 (17%) on anxiety disorders (five on Generalized Anxiety Disorder, four on panic attacks, and one on health anxiety). There were nine on suicidal ideation/suicide risk, eight on suicide attempts, six on self-harm, and two studies that assessed trauma-related disorders. Please note that multiple studies included a combination of these subjects, therefore are counted in more than one of the above outcome categories.

Most studies relied on scales or survey data based on self-reported symptoms or clinician diagnosis of symptoms or the disorder. In addition, there were ten articles that assessed rates of psychotropic medication use as a proxy for mental disorders, eight that analyzed mental health service use (community or hospital), one that assessed length of hospital admissions, and one that assessed internalizing behaviours in children.

Populations studied included all adults in 72 articles, working age adults in 36 articles, 9 studies of older adults (definitions ranged from adults >50 to >75 years old), 5 of children and adolescents, 2 that explicitly studied young adults, and 3 on middle-aged adults exclusively. 

## 4. Discussion

Our study found that there has been considerable research on symptoms of depression, anxiety, and suicide mortality associated with periods of economic recession published in English from OECD nations since 2008. Unfortunately, studies on trauma-related disorders, such as PTSD, and in special populations, including children, adolescents, and older adults, were all found to be quite limited. In addition, there has been a considerable body of research in this area representing the European continent, particularly the Mediterranean region and the United Kingdom, as well as the United States of America. Research studies completed in other OECD continental and country blocks were quite limited, as in the Asian and Commonwealth nations, and completely absent in the case of Central and South American OECD nations. No African countries are currently included among the OECD Member Nations. 

### 4.1. Depression

Following the 2008 global financial crisis (GFC), an increase in the prevalence of depressive symptoms and disorders was seen across most of the developed world. A pervasive increase in mental health care utilization for depressive symptoms was seen during or following periods of economic recession [10,11,12,13]. Among outpatients, physician visits for mental health care increased among women, those with increased age, family income, health care access/coverage, and education levels in the United States of America (USA); however, visits decreased overall during the recession for both men (25%) and women (7–8%) of all ethnic backgrounds [14,15]. Psychotropic drug use increased post-recession among USA women, USA adults in the Northeast region, USA plant workers, Italian and Spanish adults, and new mental health outpatients in Canada [14,16,17,18,19,20].

Twenty-two studies reviewed the association between unemployment and depression—twenty-one of these studies found a positive relationship between these two factors. Correlation coefficients between unemployment and depressive symptoms/disorders ranged from 0.139–0.68 in two European studies [21,22]. Countries with a higher unemployment rate post-2008 GFC compared to pre-GFC had increased likelihood and severity of depressive symptoms [23,24,25]. Furthermore, the probability of chronic mental illness was found to increase with national unemployment rates during the GFC [26].

Individual-level unemployment was found to increase depressive symptom scores by 0.6–2 points or 3.18–7.33% on the Center for Epidemiologic Studies Depression Scale (CES-D) [25,27,28,29]. Job loss during the 2008 GFC was found to increase the odds of having an incident mood disorder 1.65–2.02 times in Greece and the Netherlands, 16.6% in six European countries, or 22.5% in the USA, and in particular, job loss secondary to firm closure had an increase in depressive symptoms of 28.2% in the USA and 7.5% in Europe on the CES-D [28,30,31,32,33]. Similarly, individual level employment was found to decrease depressive symptoms across European nations and for American men during the GFC [34,35,36].

Men appear to be a particularly vulnerable group with multiple studies finding a more robust relationship between depression and unemployment for men than women [22,23,31,37,38,39,40]. Job insecurity has also been associated with increased odds of depression/depressive symptoms 1.3–1.86 times in Europe, the United Kingdom (UK), the USA, or a 33.5% increase in depressive symptoms [23,41,42]. Precarious employment was correlated with higher depressive symptoms scores on the CES-D across 21 European countries (correlation coefficient = 0.077), and a sudden decrease in workload was found to increase the probability of depressive symptoms by 8.6% [34,43,44].

Reduction in income has been associated with increases in depressive symptoms in European, South Korean, and American studies [21,27,31,32,35,45,46,47]. Reduced individual or household income has been associated with 1.77 times increase in odds of an incident mental disorder and 1.74–2.24 times or an 11.7% increase in odds of depressive symptoms/disorder [31,32,35]. Similarly, economic distress and financial strain have been found to increase depressive symptoms by nine out of ten studies assessing these measures [27,30,41,42,48,49,50,51,52]. Reporting economic distress was associated with a 1.5-point increase on the CES-D and a 1.16–1.33 times increased odds ratio of MDD [27,30,42,48,52]. Positive social support was found to be protective against the negative effects financial stress on depression, whereas interpersonal trust was only protective against MDD (5% decreased odds) for those who had low economic distress [50,51].

Housing insecurity was a significant mediating factor in depressive symptoms associated with the 2008 GFC as assessed by seven studies [18,42,53,54,55,56,57]. They found 1.2–5.8 times higher odds of MDD associated with foreclosures, 2.11 times higher odds of depressive disorders associated with mortgage payment difficulties, and 3.7 times higher odds of MDD for those behind on their rent [42,54,55,57].

Overall, life satisfaction, perceived health, eudaimonic well-being, individual optimism, social optimism, close relationships, positive social supports, becoming married, maintaining employment, and having a higher level of education were generally found to be protective against depressive symptoms during the 2008 recession [36,45,49,58].

For people with depression at baseline, preceding the 2008 GFC, they were found to have increased risk during the recession of job loss, becoming a caregiver, or having major illness personally or in a family member [52,59]. There were also 2.2 times increased odds of financial hardship during the recession associated with a 12-month history of any mental disorder that was not significantly related to change in employment, social status, or debt levels [60].

### 4.2. Anxiety

Overall levels of anxiety were found to be stable or in decline during periods of economic recession among USA and Canadian adults during the 2008 GFC and the 2015 oil recession [18,36,61]. However, among workers in particular, anxiety appears to increase during recessionary periods. In the post-recession period, an 11% increase in anxiolytic prescription was seen among USA plant workers, 7.3% increase sedative prescriptions for Portuguese men, and among workers in Spain, 69.8% of long-term sickness absence was due to anxiety disorders [16,62,63].

Studies found that during times of economic recession, both job insecurity and unemployment were associated with increased anxiety [16,32,63,64,65,66,67]. Income reduction and financial distress were not found to be consistently related to anxiety. While in the Netherlands, no association was found with incident anxiety with decreased household income and onset or recurrence of anxiety disorders across income categories during the recession, in the USA, financial strain and anxiety symptoms were found to be correlated (coefficient = 0.062), as well as in Portugal, Greece, and Spain [31,45,46,49,50,68].

In two studies on GAD, an increased odds ratio for diagnosis was seen in the USA after the 2008 GFC, associated with individuals who experienced financial impacts (odds ratio (OR) 1.3) or foreclosure (OR 1.9), as well as for people with less than college level education (OR 1.8) [42,55]. A one standard deviation increase in financial advantage conferred a 1.3 times increased risk of GAD with each negative housing impact experienced [42].

Three studies assessing symptoms of panic attacks or panic disorder were completed in the USA following the 2008 GFC [42,43,57]. They found increased odds of panic symptoms associated with experiencing a negative financial, job-related, or housing impact (OR 1.2), housing instability (OR 2.5), being behind on the mortgage or foreclosure (OR 3.7), and foreclosure in the past three years (OR 3.5) [42,57]. People who perceived job insecurity were 21.2% more likely to experience anxiety attacks compared to the job secure, and perceived insecurity plus unemployment increased risk beyond perceived insecurity alone [43].

During previous economic recessions, becoming married, having increased occupational prestige, and a higher level of education were found to be protective against anxiety disorders [45,49], whereas interpersonal and institutional trust were not correlated significantly with GAD in whole population samples, or samples of Greek adults in 2011 with low or high levels of financial strain [45,49,51]. Negative social support was correlated with increased anxiety symptoms and positive social support limited the effects of financial stress on anxiety levels [49,50].

### 4.3. Trauma-Related Disorders

Only two studies that addressed trauma-related disorders met the inclusion criteria. The first is a 2012 study of adults from Detroit, USA that found that people with a history of PTSD were at 6.2 times greater odds of foreclosure during the 2008–2010 GFC [55]. The second is a 2018 time-trend analysis of new patients assessed at mental health clinics in Fort McMurray, Canada, during the oil recession of 2015, which found that the number of new patients with trauma-related diagnoses during the recession compared to pre-recession decreased to 8.2% from 14.2% [18].

### 4.4. Self-Harm

The five articles included that studied self-harm in adults related to economic recessions all found increased rates of self-harm during or following periods of recession [18,69,70,71,72]. Characteristics associated with higher rates of self-harm included unemployment or job insecurity, financial stressors, and housing insecurity [69,71]. In Ireland, episodes of self-harm among males 31% and 22% among females were beyond the expected rates if pre-recession trends had continued. This resulted in 5029 excess hospital presentations for the treatment of self-harm in men and 3833 for women in the five-year period following the 2008 GFC [72]. In community mental health clinics in Fort McMurray, Canada, new patients with a history of self-harm increased from pre-recession rates of 13.6% of new patients to 16.6% following the 2015 oil recession [18].

### 4.5. Suicidal Ideation or Attempt

Of 12 studies on SI and attempts related to periods of economic recessions, two did not find a significant change in SI or attempt rates during/post-recession compared to pre-recession. Income inequality and personal economic distress has been associated with an increased risk of SI and attempts in South Korea and Greece [30,47,52]. Studies in Europe found that in the post-recession period people at higher risk of SI and attempt were unemployed, had financial hardship, low interpersonal trust, were married (53 times greater risk than unmarried), perceived a negative impact of the GFC, and had a history of suicide attempt (14.41 times risk) or MDD (97 times greater risk) [27,52,73,74,75,76,77,78,79]. The median age of people who attempt suicide increased following the GFC to middle-aged adults, particularly those approaching retirement [74,75,80].

### 4.6. Suicide

Across 48 studies assessing suicide mortality rates (SMR), nearly all studies found an increase in suicide rates during and following period of recession. A total of three studies in Spain, Italy, and Greece found no significant increase in SMR at a population level [81,82,83]. In a study of SMRs in the USA between 1928 and 2007, rates were found to consistently increase during recessions and decrease during expansions [84]. In some studies, a possible six month to two-year lag in increasing SMRs following the trough in economic activity has been noted [85,86,87].

In Japan, Europe, and the Americas, male SMRs were seen to increase disproportionate to female SMRs in Spain, the Netherlands, Ireland, Eastern Europe, Italy, and across a grouping of 27 European countries [72,80,88,89,90,91,92]. Overall, the 2009 male SMR across 55 countries—27 in Europe and 18 in the Americas—was increased by 3.3% (or 5124 excess suicides) [89]. The SMR for working-aged men (25–64 years) increased by 4.2–12% in European studies, while no significant change was seen for women [89,90]. Across 18 American countries, male SMRs rose 6.4% or 3175 excess suicides following the 2008 GFC, compared to a 2.3% rise among females in the Americas [89]. Other studies in the USA found that the 2008 GFC explained 30% of the change in short- and long-term SMRs observed up to 2016 [86,92].

During periods of crisis, certain characteristics were observed among people who completed suicide, with high levels of neuroticism increasing risk of suicide 2.45 times and increased levels of interpersonal trust being protective against population level suicide [93,94]. Among men, the level of education had an inverse relationship with SMRs, while no clear relationship was observed for women [92]. By age group, five studies found that people (particularly men) of working age, approaching retirement were at higher risk than other age groups [80,82,95,96,97]. Relationship status was inconsistently associated with an increased risk of suicide during a recession [82,95,97]. While mental illness remains one of the most significant risk factors for suicide during times of recession (28% to 61%), multiple studies reported no change, or a decrease in comorbid mental health diagnoses among people who died by suicide in recession times [80,95,97,98,99].

Twenty-three studies assessed SMRs in relation to job security, financial strain, and unemployment. Of these studies, five found no population level association between unemployment levels and SMRs in the USA, Spain, and Italy [82,100,101,102,103]. Suicide rates were found to increase with each 1% increase in male unemployment rates by 0.94–1.6% among men [89,94,104]. The effect of unemployment on SMR was greatest in European countries with the weakest unemployment protection, and across 55 developed nations, countries with a lower pre-crisis unemployment rate (<6.2%) showed a stronger correlation with male suicide rates [89,91]. In addition, each increase in $10 spent by governments on labour market programmes decreased the effect of a 1% increase in male unemployment on SMRs by 0.026% [91]. These findings were supported by national level studies in Australia, Belgium, England, Greece, Hungary, Spain, Sweden, and the USA [93,95,97,105,106,107,108,109,110,111,112,113,114,115].

People employed in jobs with low occupational prestige were more likely to commit suicide than high prestige jobs, including managers and supervisors [96,116,117]. Gross domestic product (GDP), markers of economic output (ICEI), and other measures of economic activity have been found to vary counter-cyclically with suicide rates in South Korea, Spain, the USA, Greece, and Europe [83,86,102,108,117,118,119,120].

Five studies reviewed the association between SMR and housing insecurity. Four studies in the USA following the 2008 GFC found a positive association between foreclosure rates and eviction [98,104,121,122]. A 1% increase in foreclosure rate was found to add 1.2 additional suicide deaths per 100,000 across the USA, or a 0.10 suicide rate/100,000 associated with a 1% increase in state-level foreclosure rate [121,122]. However, these rates were most significant for white men, and for those nearing retirement (ages 46–64) [104,121,122]. Real-estate owned foreclosure rate was found to be a stronger predictor than the total foreclosure rate, and 79% of suicides related to foreclosure occurred prior to the actual loss of housing with 37% within two weeks of a crisis related to eviction/foreclosure [98]. Eviction was found to increase odds of suicide 5.94 times among Swedish adults following the 2008 GFC [123].

### 4.7. Special Populations: Children and Adolescents

In six studies looking at the impact of economic recession on depression children and adolescents, there was evidence of correlation between early socioeconomic adversity and depressive symptoms seen in the UK, USA, Finland, and Sweden [124,125,126,127,128,129]. These changes are at least in part attributable to parental unemployment, household income, parental education level, parenting style, youth unemployment, and a perceived external locus of control in adolescence [124,126,127,128,129].

Two studies addressed anxiety related to recessions in this population, finding that youth exposed to unemployment had an increased odds ratio of anxiety in middle age and that among young adults in Portugal post-recession there was an increase in the use of prescription psychotropic drugs [62,125]. No studies were identified on trauma-related disorders among children and adolescents related to economic recession.

With regards to suicidality, one study of the pediatric population in Denmark found no effect of the GFC [130], while a USA study found that statewide job loss of 1% was related to a 2% increase in SI and a 2.2% increase in suicide plans among adolescent females and a 2.3% increase in SI, a 3.1% increase in suicide plans, and a 2% increase in suicide attempts was seen among non-Hispanic black adolescents [131]. No association was seen for adolescent males, non-Hispanic whites, or Hispanics [131]. A study focused on youth (ages 15–24) from high-income countries found that those in countries with high levels of income inequality and GDP in 2008 saw rising suicide rates among this population [132].

### 4.8. Special Populations: Older Adults

In eight studies of the impact of economic recessions on depressive symptoms in older adults, results were varied based on factors unique to this population. A study in the USA found that older adults had an increase in MDD diagnosis greater than the general population between 2005 and 2015, and 35.3% of respondents at a health centre in Greece reported that the economic crisis had provoked depressive symptoms [45,128]. For USA adults over 50 years, new food insecurity during the 2008 GFC was associated with 1.7 times odds of MDD compared to those who were food insecure at baseline [133]. For older adults with newly co-residential adult children during the 2008 recession in the USA, CES-D scores were seen to increase on average 0.179 points. If co-residential adult children were unemployed (vs. employed), the CES-D score increased an average of 0.522 points [134]. In addition, education, chronic disease presence, annual income, and a reduction in income >20% were not associated with levels of geriatric depression among respondents in Greece [45]. In contrast, two studies of 13 European countries found that retirement was protective against depressive symptoms, particularly for blue-collar workers in regions severely hit by the economic crisis [34,135].

Three studies were included that addressed the impacts of economic recessions on anxiety symptoms in older adults [62,66,136]. A prospective cohort study of older adults in Australia found that the economic slowdown during the GFC correlated with an increase in anxiety symptoms not explained by sociodemographic or economic factors [136]. Overall psychotropic drug use among older adults was not observed to change post-recession, but among female retirees and home makers post-recession in Spain, the odds ratio of sedative use increased 1.23 and 1.30 times, respectively [62,66].

No studies were identified addressing trauma-related disorders or self-harm among older adults related to economic recessions. In one cross-sectional study in Spain after the 2008 GFC, they found that adults aged 65 and older were more likely to report SI in the context of household financial problems than other age ranges surveyed [78].

Three studies were included that specified impacts of economic recession on suicides among older adults. One study found that for adults over age 65 a decrease in the ICEI was protective against suicide, and in another, an inverse relationship was seen between the foreclosure rate and suicides among adults over age 65 [104,118]. However, in contrast, a study in the Netherlands found a sudden increase in SMRs in 2007–2013, with a shift in the peak age group of suicides among men from 30–39 years to 60–69 years after 2008 and among women this shifted from 30–39 to 50–59 years old [80].

### 4.9. Limitations

The results of this study are limited by the quality and diversity of data available and included in the review. A critical appraisal of included articles was not completed as part of this scoping review. In addition, although the inclusion criteria limited studies to current quantitative evidence since 2008, published in peer-reviewed journals, and conducted in OECD countries, the data included are highly diverse and reflective of a broad spectrum of political and economic climates and policies. Therefore, these data may not be generalizable; caution should be used in interpreting findings on topics included in this study with limited data available, such as trauma-related disorders, and all topics reviewed for pediatric and geriatric populations. This study does identify considerable room for further research in these areas. Furthermore, data included are gathered at a population level and may not be generalizable to any specific individual.

This review seeks to summarize available data quantifying the mental health impacts of economic recessions but does not specifically include evidence-based interventions to manage the same; statements made in this regard are largely speculative in nature. The exclusion of qualitative research limits the ability of this review to comment on explanatory issues around mental health in economic recessions.

The basis of each economic recession studied is varied, with many studies based on recessions following an economic crisis in the housing markets or stock markets. Therefore, the specific populations impacted by the current COVID-19 pandemic, as well as rates of mental illness and suicide, may vary and risk levels among sub-populations may diverge from those seen in previous recessions.

Potential biases in this study in the search strategy include the possibility of failing to include research studies that did not address the target mental disorders and economic recessions as their primary outcome and may not have included this in the abstract of the article but did discuss relevant information. In addition, articles citing these subjects but not completing primary research in this area would return as search results utilizing this strategy. These issues were addressed by a review of all excluded articles for relevant citations in their reference lists prior. The limitation of studies to English language only significantly biases this review towards research completed in anglophone nations within the OECD and may not be representative of this entire group. In addition, articles not accessible online or through major databases were not included in this search strategy and results are therefore biased by the inclusion criteria for databases utilized. We attempted to mitigate these concerns by using a combination of both subscription-based and open-access databases and search engines. Finally, there is a risk of reviewer bias inherent in this type of study, which was diminished by utilizing two reviewers in the article review process.

## 5. Conclusions

Overall, the results of this scoping review suggest that general models for providing mental health support and strategies for suicide prevention may be less effective in reaching those whose mental health is negatively impacted by an economic recession than they are in non-recession times [14,15,80,95,97,98,99]. The populations found by most studies to primarily be effected by depression, self-harm, and suicide secondary to economic recession include men approaching retirement age, people with low education, high levels of unemployment or job insecurity, and low pre-recession socioeconomic status, yet those most likely to access mental health supports were found to be women and highly educated adults [14,15]. Additionally, analyses of the characteristics of people who committed suicide related to economic recession found that they were less likely to access the family physician or mental health supports, or be formally diagnosed with mental illness, prior to suicide compared to non-recession related suicides [80,95,97,98,99]. Therefore, it may be prudent to focus public educational efforts to increase vigilance to identify people in need of support at a community level, among places of employment, unemployment, or income support offices. For healthcare providers, people presenting with complaints of mental health concerns during recession times, particularly those in the higher risk groups outlined above, should be carefully evaluated for SI and safety; and perhaps a lower threshold for treatment may be warranted.

At a governmental level, policy makers should be aware of the potential protective nature of unemployment protections and labour program investment in mitigating the negative impacts of economic recession on population level mental health and suicide mortality [89,91,94]. However, increasing resources during times of recession is inherently challenging, as governments are limited in their ability to invest in new mental health supports by the economic reality of a recession. As such, during recessions, governments typically lay off staff, do not replace retiring staff, or avoid creating new heavily human resource intensive mental health services [137,138]. Therefore, health policy and practice implications should also consider the adoption of low cost, evidence-based interventions such as bibliotherapy, Internet-Delivered Cognitive Behavioural Therapy, supportive text messaging, and encouragement of community and family level emotional support [138,139,140,141,142,143,144,145,146,147,148,149,150,151,152,153,154,155,156,157,158,159,160,161,162]. Research evidence has suggested that support from family and friends is protective during natural disasters and two studies in this review suggest that positive social support provides additional protection against anxiety and depression during times of economic recession [49,50]. Therefore, study of low-cost interventions for mitigation and treatment of mental health concerns during periods of economic recession could be a tremendously beneficial area for future study.

## Figures and Tables

**Table 1:** Summary of included articles on depression only (N = 30).

Author & Date	Country of Study	Study Type	Recession Studied	Mental Health Outcomes	Key Findings
Longworth Swift, S., Elfassy, T., Bailey, Z., et al. (2020)	USA	Prospective Cohort Study	2008 GFC	Depressive sx (CES-D)	Unemployed (8.3%) scored 2 points higher on CES-D. Income drop (15.2%) scored 1.1 point higher on CES-D. Experience of a negative shock (debt > assets; 6.7%) scored 1.5 points higher on CES-D.
Hammarstrom, A., Virtanen, P. (2019)	Sweden	Prospective Cohort Study	1980s	Depressive sx	Lower depressive sx at baseline (age 21) were seen among the boom cohort compared to the recession cohort. At follow-up, the difference in depressive sx scores was not significant due to decreased sx in the recession cohort.
Pruchno, R., Heid, A.R., Wilson-Genderson, M. (2017)	USA	Prospective Cohort Study	2008 GFC	Depressive sx (CES-D)	A significant increase seen in mean depressive sx overall between baseline and follow-up (t = −24.93). Those with no depression at follow up vs. incident depression had higher levels of income, education, and were less likely to be married at baseline. They were less likely to have lost a job, become a caregiver, personally experience or had a family member who experienced a major illness at follow-up.
sCulpin, I., Stapinski, L., Budanur Miles, O., et al. (2015)	UK	Prospective Cohort Study	early socio-economic adversity (non-specific)	Depression at age 18	Greater early socioeconomic adversity was associated with an increased risk of depression at 18 years (β = 0.191). Evidence of an indirect path from early social adversity through locus of control to diagnosed depression at 18 years (β = 0.128) accounting for 34% of the total estimated association.
Thekiso, T.B., Heron, E.A., Masood, B., et al. (2013)	Ireland	Prospective Cohort Study	2008 GFC	Depressive sx (LIFE-PSR for Major Depression)	100% of inpatients with depression secondary to GFC (“Celtic Tiger” group) achieved full recovery post discharge compared to 79% of the control group. 12% had at least one full recurrence compared to 56% of the control patients. Mean time to recurrence was 696 days for the Celtic Tiger group versus 405 days for the control group. The Celtic Tiger group had slower time to partial recurrence than controls and fewer had a partial recurrence (12% vs 44%).
Wang, Y., Fattore, G. (2020)	Italy	Time Trend Analysis	2008 GFC	Affective Disorders (bipolar or MDD)	A 1% increase in unemployment gives rise to about 1 out of 100,000 residents being admitted to the hospital due to an affective disorder. None of the other variables were found to have a statistically significant relationship with admission rates.
Bergmans, R.S., Wegryn-Jones, R. (2020)	USA	Time Trend Analysis	2008 GFC	Major Depression (CIDI-SF)	Compared to those who were not food insecure, those who were initially food insecure had 1.2 times greater odds of MDD, those who became or remained food insecure had 1.7 times greater odds of MDD. Becoming food insecure was associated with major depression in the time periods during and immediately following the Great Recession but not in later years.
Todd, M., Teitler, J. (2019)	USA	Time Trend Analysis	2008 GFC	Depressive sx (PHQ-9)	Depression was greater in the least vs. the most educated group for both sexes, with women having more depressive sx than men. Among depressed people, 80% of college educated people received therapy/rx, vs. 55–75% of those with <12 years of education. Disparities persisted or worsened throughout this period.
Caputo, J. (2019)	USA	Time Trend Analysis	2008 GFC	Depressive sx (CES-D)	Older adults with a newly co-resident adult child between 2008–2010 reported CES-D scores 0.179 points greater than those without, transitioning to co-residence with an out-of-work vs. employed child was associated with a 0.522-point increase.
Chaves, C., Castellanos, T., Abrams, M., et al. (2018)	Spain	Time Trend Analysis	2008 GFC	Depressive sx (CES-D)	CES-D scores increased from 2006–2007 to 2013 (5.60 to 6.07), while life satisfaction decreased (7.45 to 6.94). All variables negatively correlated with depressive sx as follows: Perceived physical health (−0.40), individual optimism (−0.37), life satisfaction (−0.47), eudaimonic well-being (−0.50), close relationships (−0.24), social optimism (−0.20), and social trust (−0.09).
Rodrigues, D.F.S., Nunes, C. (2018)	Portugal	Time Trend Analysis	2008 GFC	Hospital admissions for MDD	An increase in relative frequency of hospitalization was seen for males between 2008 to 2013 (0.3% to 0.4%), but for females it remained stable at 0.5%. National average hospitalization rates for MDD were 25.60 in 2008 and 26.17 in 2013 per 100,000 of the working-age population.
Weinberger, A.H., Gbedemah, M., Martinez, A.M., et al. (2017)	USA	Time Trend Analysis	2008 GFC	Past year and lifetime MDD	The prevalence of past-year depression increased from 2005 to 2015 (6.6% to 7.3%). Increases observed among 50+ and significantly more rapid increase among youth ages 12–17 than every other group, particularly after 2008. No significant gender difference. An increase was seen among lowest and highest annual household income groups.
Pelekasis, P., Kampoli, K., Vtavatzikos, A., et al. (2017)	Greece	Time Trend Analysis	2008 GFC and Austerity Measures	Depressive sx (IDS-C30)	The 2015 patient group had fewer depressive sx with an effect size, r, of 0.53. The strongest effect size was reported for those who were divorced (r = 0.80), widowed (r = 0.74) and had 3+ children (r = 0.71). The lower effect size was reported for those who were non-Greek (r = 0.14), having two children (r = 0.38), not cohabitating or 70–80 y.o. (r = 0.40).
Axelrad, H., Sabbath, E.L., Sherburne Hawkins, S. (2017)	13 European Countries	Time Trend Analysis	2008 GFC	Depressive sx (EURO-D)	No effects of GDP, life expectancy, or country-level unemployment on depressive sx were seen. Individual level employment was found to decrease depressive sx (B 0.36, SE 0.07, *p* < 0.01) and retiring during the follow-up period (all participants were employed at the first interview) was associated with fewer depressive sx (B 0.40, SE 0.07, *p* < 0.01).
Reibling, N., Beckfield, J., Huijts, T., et al. (2017)	21 European Countries	Time Trend Analysis	2008 GFC	Depressive sx (CES-D)	Most countries reported fewer depressive sx in 2012 vs. 2006. Countries with a higher GDP per capita before the crisis exhibit a lower level of depressive sx (Beta = 0.008). Individuals were more depressed with a lower income (Beta = 0.122), inactive employment (Beta = 0.099), when unemployed (Beta = 0.139), or precariously employed (Beta = 0.077); and when income is primarily from public benefits (Beta = 0.098).
Rodrigues, A.P., Sousa-Uva, M., Fonseca, R., et al. (2017)	Portugal	Time Trend Analysis	2008 GFC	Depression rates per 100,000	An increase in the depression incidence rate was seen starting in 2004 for both genders. 83% of the variability in depression was related to the unemployment rate in males. In females, this was not statistically significant. Suggest an increase of 37 depression cases per 100,000 per 1% increase in male unemployment.
Economou, M., Angelopoulos, E., Peppou, L.E., et al. (2016)	Greece	Time Trend Analysis	2008 GFC	MDD	Overall women manifest higher rates of major depression than men, irrespective of age. However, in 2013, as Greek recession deepened, men of productive age became increasingly more vulnerable to major depression (ages: 18–24, 35–44, and 45–54 y.o.).
Belloni, M., Meschi, E., Pasini, G. (2016)	10 European Countries	Time Trend Analysis	2008 GFC	Depressive sx (EURO-D)	Retirement improved mental health in periods and regions severely hit by the economic crisis. This effect was entirely due to blue-collar (ex)workers and did not apply to white-collar workers.
Buffel, V., Van de Velde, S., Bracke, P. (2015)	20 European Countries	Time Trend Analysis	2008 GFC	Depressive sx (CES-D)	In countries with a high increase in the unemployment rate from 2005 to 2011, women (b = 0.047) and especially men (b = 0.053) had a higher likelihood of being depressed. The crisis effect was significantly stronger among men and more pronounced for those 35–49 y.o. and remained significant for both men and women even when individual employment status was accounted for. Employed men and women with a limited contract and employed men with no contract have a higher likelihood of reporting depressive sx compared with the employed with an unlimited contract.
Kendrick, T., Stuart, B., Newell, C., et al. (2015)	England	Time Trend Analysis	2008 GFC	Non-psychotic depression	For males, the analysis showed a significant increase in the prevalence of depression. Prior to quarter 2 of 2008, there was a negative correlation between overall prevalence and unemployment (Pearson’s rho −0.48), but after this time the correlation was moderately positive (0.68); this association was greater among males than females.
Drydakis, N. (2015)	Greece	Time Trend Analysis	2008 GFC	Depressive sx (CES-D)	Unemployment during the 2008–2009 period entails a negative unemployment effect on mental health of 4.64% for women and 3.18% for men. Unemployment during the 2010–2013 period entails a negative effect on mental health of 7.33% for women or 4.93% for men.
Park, J.E., Lee, J.Y., Sohn, J.H., et al. (2015)	South Korea	Time Trend Analysis	2008 GFC	MDD (CIDI)	The prevalence rate of MDD in 2011 vs. 2001 increased for men by 4.19 and 1.39 for women. A significant increase was observed in unemployed men (AOR 8.35), but not in unemployed women.
Riumallo-Herl, C., Basu, S., Stuckler, D., et al. (2014)	13 European Countries & USA	Time Trend Analysis	2008 GFC	Depressive sx (CES-D)	After controlling for household wealth, household income, pension receipt, health behaviour, and functional status, job loss was associated with a 4.78% increase in depression scores in the USA and a 3.35% increase in Europe. Job loss due to firm closure increased depressive sx scores by 28.2% in the USA and by 7.50% in Europe.
Cagney, K.A., Browning, C.R., Iveniuk, J., et al. (2014)	USA	Time Trend Analysis	2008 GFC	Depressive sx (CES-D)	For all 3 stages of the foreclosure process, residing in an area that underwent an increase in housing stock foreclosure increased the risk of developing depressive sx (for notices of default, OR = 1.75; for auctions, OR = 1.45; for real-estate owned, OR = 1.62).
Torikka, A., Kaltiala-Heino, R., Rimpela, A., et al. (2014)	Finland	Time Trend Analysis	2008 GFC	Depression (Finnish BDI)	Severe depression peaked among girls in 2010–2011 and among boys in 2008–2009. Among boys and girls whose parents had a low education level and were unemployed, severe depression was reported by 6.5% and 6.4% respectively in 2000–2001 and by 12.8% and 11.4% respectively in 2010–2011.
Economou, M., Madianos, M., Peppou, L.E., et al. (2013)	Greece	Time Trend Analysis	2008 GFC	MDD (SCID-I)	One month prevalence 8.2% (2011) vs. 3.3% (2008); OR of suffering MDD in 2011 vs. 2008 is 2.6. Every unit increase in the Index of Personal Economic Distress score was found to increase the odds of suffering from major depression by 1.2 (OR = 1.2).
Tapia Granados, J.A., Christine, P.J., Ionides, E.L., et al. (2018)	USA	Cross-sectional Study	2008 GFC	Depressive sx (CES-D)	Being unemployed was significantly associated with higher levels of depressive sx (0.60 mean increase in CES-D score.) A 1% increase in the unemployment rate was associated with a 0.23-unit increase in the CES-D score of depressive sx.
Brenner, M.H., Andreeva, E., Theorell, T., et al. (2014)	France, Hungary, Sweden, and the UK.	Cross-sectional Study	2008 GFC	Depressive sx (Hopkins sx Checklist)	Odds of suffering from depressive sx were 2.85 in persons unemployed at interview and 2.04 in layoff survivors, as compared to the re-employed. Depressive sx were increased if workers perceived downsizing as chaotic (OR 2.5), had diminished income and benefits (OR 1.74), and among layoff survivors with early warning of layoffs (OR 0.46). Early warning was protective for those laid off (OR 2.13).
Modrek, S., Cullen, M.R. (2013)	USA	Cross-sectional Study	2008 GFC	Depression	2009–2010: 1.1% of the workforce was diagnosed with depression. No association between working at a high layoff plant and acquiring a new diagnosis of depression, and no association between downsizing and depression.
Meltzer, H., Bebbington, P., Brugha, T., et al. (2010)	Great Britain	Cross-sectional Study	2008 GFC	MDD (Clinical Interview Schedule)	20% of all working men and women aged 16–64 y.o. felt that their job security was poor. After controlling for age and sex, job insecurity (OR 1.86) and being in debt (OR 2.17) were independently associated with depression.

Abbreviations in Table 1: Beck Depression Inventory (BDI); Center for Epidemiologic Studies Depression Scale (CES-D); Composite International Diagnostic Interview—Short Form (CIDI-SF); European Depression Scale (EURO-D); gross domestic product (GDP); Global Financial Crisis (GFC); The Inventory of Depressive Symptomatology, Clinician Rating, 30-item (IDS-C30); Longitudinal Interval Follow-up Evaluation Weekly Psychiatric Status Rating Scale (LIFE-PSR); Major Depressive Disorder (MDD; odds ratio (OR); Personal Health Questionnaire—9-item (PHQ-9); medications (rx); Structured Clinical Interview for DSM-IV Axis I Disorders (SCID-I); symptom(s) (sx); United Kingdom (UK); United States of America (USA); versus (vs); years old (y.o.).

**Table 2:** Summary of included articles on depression, anxiety, and post-traumatic stress disorder (N = 36).

Author & Date	Country of Study	Study Type	Recession Studied	Mental Health Outcomes	Key Findings
Virtanen, P., Hammarstrom, A., Janlert, U. (2016)	Sweden	Prospective Cohort Study	1980s Recession	Depressive sx and anxiety sx	Exposure to youth unemployment was associated with an upper quartile score of anxiousness in middle age (OR 2.19 for boom cohort, and OR 2.13 for recession cohort). High exposure to unemployment in youth predicted depressiveness in middle age in the boom cohort (OR = 1.85) but not in the recession cohort (OR = 1.38); the difference between groups was insignificant.
Barbaglia, M.G., ten Have, M., Dorsselaer, S., et al. (2015)	The Netherlands	Prospective Cohort Study	2008 GFC	Mood and anxiety disorders (CIDI 3.0)	Household income reduction was associated with incident mental disorders (aOR = 1.77). Job loss and household income reduction increased the risk of an incident mood disorder after 3 years (aOR = 2.02 and aOR = 2.24, respectively). Job loss was more correlated with an incident mental disorder among men (aOR = 3.04) and household income reduction among women (aOR = 2.32). No association observed for incident anxiety.
Dijkstra-Kersten, S.M.A., Biesheuvel-Leliefeld, K.E.M., van der Wouden, J.C., et al. (2015)	The Netherlands	Prospective Cohort Study	2008 GFC	Depressive and Anxiety Disorders	Participants with financial strain at baseline were not more likely to become depressed or anxious during follow-up. Post hoc analysis stratified by income levels showed comparable results: Financial strain was not related to the onset/recurrence of depressive and/or anxiety disorders in any income category.
Sargent-Cox, K., Butterworth, P., Anstey, K.L. (2011)	Australia	Prospective Cohort Study	2008 GFC	Goldberg Depression and Anxiety Scales	Those who indicated the economic slowdown impacted them had a larger increase in depression and anxiety sx. A significant increase in sx during the GFC period was not explained by demographic or socio-economic factors such as an increase in financial hardship over time.
Barcelo, M.A., Coll-Negre, M., Coll-de-Tuero, G., et al. (2016)	Spain	Prospective Cohort Study	2008 GFC	Prescription psychotropic rx use	Psychotropic rx consumption increased in 2009–2012 vs. 2005–2008 (1.194 drugs/individual/month and DDD 0.246 vs. 1.162 drugs/ind/mo., DDD 0.177). There was a statistically significant increase in the probability of being unemployed for increases in both the number of rx and the DDD prescribed.
Real, E., Jover, L., Vergaguer, R., et al. (2016)	Spain	Retrospective Cohort Study	2008 GFC	Duration of sickness absence	Most sickness absences were due to anxiety disorders (4963, 69.8%) and ended because of clinical improvement (6541, 92%); in most cases no specialist psychiatric assessment was required (6091, 85.6%).
Schneider, W., Waldfogel, J., Brooks-Gunn, J. (2015)	USA	Retrospective Cohort Study	2008 GFC	Internalizing behavior	Worsening Consumer Sentiment Index is associated with a significant increase in internalizing behaviors (anxious, depressive, and somatic sx) among boys living with a single mother (coefficient 0.11, *p* < 0.05). Measures of parenting, household income, and maternal unemployment mediates the interaction.
Silva, M., Antunes, A., Azeredo-Lopes, S., et al. (2020)	Portugal	Time Trend Analysis	2008 GFC	Psychotropic rx use	Population use of any psychotropic rx increased 6.74% from 2009 to 2015: 3.75% among women, but no significant change among men. A 7.30% increase in sedative/hypnotic rx among men vs. no significant change among women. Adults 18–49 years were more had a 9.85% increase in all psychotropics vs. >50 years (no significant change).
Forbes, M.K., Kreuger, R.F. (2019)	USA	Time Trend Analysis	2008 GFC	MDD, GAD and panic sx	Recession impacts were associated with new MDD (financial OR = 1.2; job-related OR = 1.3; housing OR = 1.3), GAD (financial OR = 1.3), and panic (financial OR = 1.2; job-related OR = 1.2; housing OR = 1.2). Having less than a college education had 1.8× odds of GAD sx, and +1 SD in financial advantage had 1.3× odds of GAD sx with each housing impact.
Antunes, A., Frasquilho, D., Azeredo-Lopes, S., et al. (2018)	Portugal	Time Trend Analysis	2008 GFC	Anxiety and mood disorders (WHO CIDI)	Participants with any 12-month mental disorder in 2008–2009 had OR 2.20 of reporting financial hardship vs. those without. The associations between the change in employment situation, subjective social status, or debt-related financial hardship and presence of any 12-month mental disorder in 2008-2009, were not statistically significant.
Arroyo, E., Cabrera-Leon, A., Renart, G., et al. (2018)	Spain	Time Trend Analysis	2008 GFC	Psychotropic rx use	The financial crisis did not change the probability of taking antidepressant or sedative rx. Sedative consumption in individuals on short-term unemployment increased for men (OR 1.1) and women (OR 1.52) during the recession. For the retired and for home makers, women’s sedative use increased (OR 1.23 and 1.3), while men’s use decreased (OR 0.94 and 0.69).
Ritchie, A., Hrabok, M., Igwe, O., et al. (2018)	Canada	Time Trend Analysis	2015 Oil Recession	DSM-5 diagnosis	During the recession, help was sought by a higher proportion of males (45.3% vs. 38.6% pre-recession); homeowners (55% vs. 47%); and unemployed people (29% vs. 24%). A higher proportion took a psychotropic medication (67% vs. 60%). Higher level of personality disorders (12% vs. 3%) and ‘other’ diagnoses (17% vs. 10%) were made, with lower levels of depressive disorders (32.8% vs. 36.8%), anxiety (18.5% vs. 19.6%), and trauma-related (8.2% vs. 14.2%) diagnoses. The differences between suicidal sx during the recession were not significant. Incidence of people with a history of self-harm increased to 16.6% from 13.6% pre-recession.
Medel-Herrero, A., Gomez-Beneyto, M. (2017)	Spain	Time Trend Analysis	2008 GFC	Psychiatric hospitalization rates	In the post-recession period, a 13.3% increase was found for all psychiatric conditions above the trend expected from the preintervention time series. This change was accounted for by adults ages 15–34, among whom an increase of 51.6% per month for depression was seen. The relationship was not statistically significant for any other psychiatric pathology.
Chen, J., Dagher, R. (2016)	USA	Time Trend Analysis	2008 GFC	Psychotropic rx use & physician visits	Prescription drug use increased in 2008–2009 for females (IRRs = 1.20) and physician visits demonstrated a similar trend. These measures were not significant among the male population. Age, education, family income, health care access, and insurance coverage were positively associated with mental health care use. For all ethnic groups lower rates of physician visits occurred during the recession: −7–8% among females and −25% among males.
Wilkinson, L.R. (2016)	USA	Time Trend Analysis	2008 GFC	Anxiety (Beck Anxiety Inventory) & depressive sx (CES-D)	Financial strain had a significant positive correlation with anxiety sx (coefficient = 0.062). Becoming married was negatively associated with anxiety, whereas negative social support contributed to increases in anxiety. Financial strain had a significant positive correlation with depressive sx (coefficient = 0.140). Becoming married and having positive social support exerted a protective effect against depressive sx, whereas increased negative social support was associated with increased depressive sx.
Barr, B., Kinderman, P., Whitehead, M. (2015)	England	Time Trend Analysis	2008 GFC	Depressive and anxious sx, anti-depressant use	The prevalence of depressive and anxious sx in low educated men and women was markedly higher than amongst high educated groups (approx. 2-fold difference) and from 2008–2013 the absolute difference increased by 1.29% for women and 1.36% for men. The proportion of the 18–59 y.o. population out of work with a mental illness increased by 1% after 2007, or an additional 356,000 people across England. An additional 1.6 suicides and the prescription of 3715 antidepressants per 100,000 people reporting mental health problems was found in hardest hit areas.
Schaller, J., Huff Stevens, A. (2015)	USA	Time Trend Analysis	2008 GFC	Anxiety and depression	The probability of depression or anxiety rises by 22.5% following job loss. There is no evidence of significant changes in the incidence of depression or anxiety following the loss of health care insurance coverage.
Dagher, R.K., Chen, J., Thomas, S.B. (2015)	USA	Time Trend Analysis	2008 GFC	Depression or anxiety d.o.	No significant differences across the pre-/during/post-recession in depression or anxiety diagnoses for females. Males had 2% fewer diagnoses of depression during the recession and 1% after the recession, compared to pre-recession. Among the employed, males had lower odds of being diagnosed with depression during and after the recession. Differences for males with anxiety diagnoses across the three time periods were not statistically significant.
Modrek, S., Hamad, R., Cullen, M.R. (2015)	USA	Time Trend Analysis	2008 GFC	ICD-9 psychiatric codes & psychotropic rx use	The number of outpatient visits with a mental health-related diagnosis suggested a statistically significant increase. A 13% increase in antidepressant use was seen overall and use of antidepressants was significantly higher in high layoff plants than in other plants. The difference in the trend for sleep aids was driven by a small increasing trend in the use of sleep aids in the post-recession period and a large decreasing trend before the recession. The 11% increase in use of anxiolytics was almost 5 times the prerecession trend.
Bradford, W.D., Lastrapes, W.D. (2014)	USA	Time Trend Analysis	2008 GFC	Psychotropic rx use	Large countercyclical responses of mental health drug prescriptions were seen only in the northeast region, and large and statistically significant countercyclical responses of total prescriptions and doctor visits to national employment was seen across regions.
Lo, C.C., Cheng, T.C. (2014)	USA	Time Trend Analysis	2008 GFC	Chronic mental illness	Increased probability of chronic mental illness was associated with recession, female gender, and being Black (vs. White), Hispanic (vs. White), or White (vs. Asian). The contextual variable recession explained 40% of the variance in adjusted probability sample-wide and 27% of the variance in that probability within the Black subsample.
Gili, M., Roca, M., Basu, S., et al. (2013)	Spain	Time Trend Analysis	2008 GFC	Depressive disorders, GAD, and panic disorder	Between 2006 and 2010, the greatest percentage point rise in frequency was for major depression (19.4% increase) and dysthymia (10.8%). About 3.1% of the risk of having major depression could be attributed to unemployment. Additional risk was associated with mortgage payment difficulties (OR = 2.11), which accounted for 11.0% of the population risk of depression.
McLaughlin, K.A., Nandi, A., Keyes, K.M., et al. (2012)	USA	Time Trend Analysis	2008 GFC	Current sx of MDD (PHQ-9), GAD (GAD-7), & history of MDD, GAD, or PTSD.	Individuals with a lifetime history of PTSD had greater odds of experiencing foreclosure than those without a history of the disorder (OR 6.2). Foreclosure was associated with 2.4 times increase in sx of depression at the follow-up and a 1.9 times increased rate of sx of GAD.
Shi, Z., Taylor, A.W., Goldney, R., et al. (2011)	Australia	Time Trend Analysis	2008 GFC	Anxiety, MDD, or stress-related diagnosis	Unemployed people had higher rates of mental health diagnoses than all other employment categories, except in the case of stress. Those in full-time employment had a statistically significant decrease in anxiety levels, while part-time work significantly increased anxiety levels. The prevalence of current mental health treatment decreased in the full-time employed. There was no significant difference in the prevalence of SI and depression.
Codagnone, C., Bogliacino, F., Gomez, C., et al. (2020)	Italy, Spain, & the UK	Cross-sectional Study	2008 GFC	Acute stress disorder, anxiety, and depression (SASRQ)	Mental health problems predicted by economic vulnerability and negative economic shock are 41.5% in Italy, 45.8% in Spain, and 41.8% in the UK. Stress had a significant negative correlation with a higher income level (rho = −0.04), owning a house (rho = −0.08), having a larger house space (rho = −0.10), and having financial buffer stock (rho = −0.18). Stress had a significantly positive correlation with being unemployed (rho = 0.09), increased household size (rho = 0.10), having children of school age (rho = 0.12), having faced negative events (rho = 0.38), and having suffered job or income losses (rho = 0.19).
Norberto, M.J., Rodriguez-Santos, L., Caceres, M., et al. (2020)	Spain	Cross-sectional Study	2008 GFC	DSM-IV diagnosis	Distribution of diagnoses: Adjustment disorder (33.1%), mood disorders (19.1%), and anxiety disorders (14.4%). 77.3% of patients seen were unemployed or inactive. Significant relationship between adjustment disorder and unemployment in adults 30–40 y.o., whereas anxiety disorders were most frequent in employed patients.
Witteveen, D., Velthorst, E. (2020)	6 European countries	Cross-sectional Study	COVID-19 Pandemic	Sx of depression and health anxiety	Incidence rates during March and April of 2020 were depressive sx (26.2%) and health anxiety sx (37.5%). A 2.9% increase chance of avoiding anxiety with 10-point increase on occupational prestige scale. Probability of depressive sx was 8.6% greater after sudden decrease in workload, 11.7% greater with income loss vs. stable income during the lockdown, and 16.6% greater with job loss vs. job retention.
Bernal-Solano, M., Bolivar-Munoz, J., Mateo-Rodriguez, I., et al. (2019)	Spain	Cross-sectional Study	2008 GFC	Anxiety and depression	People in the legal stage of foreclosure/eviction were more than twice as likely to visit an emergency room (OR 2.36) and almost twice as likely to use psychotropic medications (OR 1.88) as compared to those in the initial stages. When the eviction was due to family problems, the probability of having anxiety, depression or stress was greater than if due to loss of employment or drop in income (OR 6.51).
Viseu, J., Leal, R., Neves de Jesus, S., et al. (2018)	Portugal	Cross-sectional Study	2008 GFC	Sx of anxiety and depression	The effect of economic hardship on anxiety and depression, and of financial threat on anxiety ceases to be statistically significant when individuals perceive social support. The relationship between financial wellbeing and stress, anxiety, and depression is stronger when individuals have a good social support.
Stavrou, G., Paikousis, L., Jelastopulu, E., et al. (2016)	Greece	Cross-sectional Study	2008 GFC	Anxiety and depression (HADS) or depression (GDS)	Patients 18–65 y.o. (HADS): An increase in annual income category associated with 0.64 units less anxiety. Every additional level of education was associated with 1.18 units less anxiety, and 1.44 units less depression. A reduction in income more than 35% was associated with an increase in depression by 1.74 units. Patients >65 y.o. (GDS): 35.3% reported that the economic crisis was the provoking factor of their depressive sx. Education, the presence of chronic disease, annual income, and the reduction in the people’s salaries beyond the mean reduction level of 20% were not associated with GDS score.
Navarro-Mateu, F., Tormo, M.J., Salmeron, D., et al. (2015)	Spain	Cross-sectional Study	2008 GFC	DSM-IV disorders—anxiety, mood	Those exposed to 3 stressful economic events during the last 12 months had 7 times greater risk of any mental disorder post-recession, particularly anxiety disorders. Economic crisis was found to effect people from all population subgroups, regardless of social standing and occupational status. However, there was an increasing risk for any disorder, any mood, and any anxiety disorder with lower income levels.
Vittadini, G., Beghi, M., Mezzanzanica, M., et al. (2014)	Italy	Cross-sectional Study	2008 GFC	Psychotropic rx use	The percentage of psychotropic rx users increased from 3.37% in 2007 to 4.08% in 2011. The number of people who used antidepressants increased 26.5%. In 49.4% of the patients, the use of psychotropic rx was limited to 1 year.
Economou, M., Madianos, M., Peppou, L.E., et al. (2014)	Greece	Cross-sectional Study	2008 GFC	Depression and GAD (SCID-I)	Interpersonal and institutional trust have a protective effect against major depression. For those in low economic distress, every unit increase in the interpersonal trust scale reduces the odds of suffering from major depression by 5% and every unit increase in the institutional trust scale reduces the odds of suffering by major depression by 6%. For those with high economic distress, interpersonal trust does not bear an association with major depression. Interpersonal and institutional trust were not found to bear a significant association with the presence of GAD.
Burgard, S.A., Seefeldt, K.S., Zelner, S. (2012)	USA	Cross-sectional Study	2008 GFC	Depression (PHQ-9) and anxiety attacks	Individuals with housing instability had 2.5 times odds of a recent anxiety attack vs. those without. Those who experienced homelessness had 0.61 times odds of depression than those with no housing instability. Those behind on their rent had 3.7 times the odds of depression as those without instability. Being behind on one’s mortgage or in foreclosure was associated with 3.7 times odds of a recent anxiety attack vs. mortgage holders with stability. A foreclosure in the past three years led to 5.8 times odds of depression and 3.5× odds of a recent anxiety attack vs. their stably housed counterparts.
Burgard, S.A., Kalousova, L., Seefeldt, KS. (2012)	USA	Cross-sectional Study	2008 GFC	Depression (PHQ-9) & anxiety attacks	17.5% perceived job insecurity and were more likely to report increased sx of depression (40.8% vs. 7.3%), or an anxiety attack (30.0% vs. 9.8%) in the past 4 weeks compared to the job secure. Respondents who reported both perceived insecurity and unemployment were significantly more likely to report depressive sx and anxiety attacks than those who only perceived job insecurity.
Wang, J.L., Smailes, E., Sareen, J., et al. (2010)	Canada	Cross-sectional Study	2008 GFC	Mental disorders (WHO CIDI)	The 12-month prevalence of MDD increased from 5.1% pre-2008 to 6.8% and 7.6% in subsequent intervals. The lifetime prevalence of dysthymia was 0.4%, 0.7%, and 1.5% in the three intervals. There were no differences in the estimated 12-month prevalence of anxiety disorders. The prevalence of MDD increased more among participants who were married or in a common-law relationship, and in participants who had a university education or higher.

Abbreviations in Table 2: Center for Epidemiologic Studies Depression Scale (CES-D); Composite International Diagnostic Interview (CIDI); Diagnostic and Statistics Manual (DSM); Generalized Anxiety Disorder (GAD); Generalized Anxiety Disorder 7-item Scale (GAD-7); Geriatric Depression Scale (GDS); Global Financial Crisis (GFC); Hospital Anxiety and Depression Scale (HADS); International Classification of Diseases, 9th Revision (ICD-9); incident rate ratio (IRR); Major Depressive Disorder (MDD); odds ratio/adjusted odds ratio (OR/aOR); Personal Health Questionnaire—9-item (PHQ-9); Posttraumatic Stress Disorder (PTSD); medication(s) (rx); Structured Clinical Interview for DSM-IV Axis I Disorders (SCID-I); suicidal ideation (SI); symptom(s) (sx); versus (vs); World Health Organization (WHO); years old (y.o.).

**Table 3:** Summary of included articles on suicidal ideation and self-harm (N = 15).

Author & Date	Country of Study	Study Type	Recession Studied	Mental Health Outcomes	Key Findings
Christodoulou, C., Efstathiou, V., Michopoulos, I., et al. (2017)	Greece	Case-control study	2008 GFC	Hopelessness (BHS) and suicide attempt	Suicide attempters presented higher hopelessness than the controls (BHS 9 vs. 3, respectively). The unemployed presented higher hopelessness than the non-unemployed (BHS 4 vs. 3). Participants with a low self-reported financial status (BHS 6) had higher hopelessness than those with a high (BHS 2) and a very high status (BHS 1). No significant difference in BHS scores before and after the GFC was seen in the two healthy participant samples.
Clements, C., Hawton, K., Geulayov, G., et al. (2019)	England	Time Trend Analysis	2008 GFC	Self-harm	The 2008–2013 cohort showed an increase in self-injury (men OR 1.62; women OR 1.60), higher unemployment (men OR 1.67; women OR 1.58) and an increase in psychiatric care (men OR 1.57; women OR 1.40). Precipitating problems included issues around employment, finances and housing.
Steeg, S., Carr, M.J., Mok, P.L.H., et al. (2019)	Denmark	Time Trend Analysis	2008 GFC	Self-harm	For adolescent boys and girls, national self-harm incidence rate increased gradually from 2000 to 2007, then from 2008 to 2016 decreased to just below the rate observed at the start of the study period. The same pattern was seen within each parental income tertile. This coincides with the introduction of nationally available psychosocial therapy for people at risk of suicide in Denmark in 2007. No effect of the economic recession in 2008 was observed.
Geulayov, G., Kapur, N., Turnbull, P., et al. (2016)	England	Time Trend Analysis	2008 GFC	Self-harm and suicide	The annual rates of self-harm appeared to decline between 2000 and 2008 for males (IRR 0.96) and then steadily increase thereafter (IRR 1.05) and among females, the rate declined until 2009 and then level off until the end of the study period in 2012. Rates of self-harm from this study were strongly correlated with suicide rates in England in males (r = 0.82) and females (r = 0.74).
Hawton, K., Bergen, H., Geulayov, G., et al. (2016)	England	Time Trend Analysis	2008 GFC	Self-harm	Increases in 2008–2010 vs. 2005–2007 were seen among those identified as having problems at the time of self-harm related to employment (M: χ^2^ = 52.5; F: χ^2^ = 35.3), finances (M: χ^2^ = 7.5; F: χ^2^ = 16.2) and, in females only, housing (χ^2^ = 7.0). Among those employed, there was an increase in males with problems related to employment (χ^2^ = 20.7) and females with employment (χ^2^ = 14.5), financial (χ^2^ = 7.0) and housing (χ^2^ = 6.0) problems in 2008–2010 vs. 2005–2007.
Cordoba-Dona, J.A., San Sebastian, M., Escolar-Pujolar, A., et al. (2014)	Spain	Time Trend Analysis	2008 GFC	Suicide attempt rates	Between 2008 and 2012 there was an excess of 4989 suicide attempts—2017 in men and 2972 in women—compared to pre-crisis historical trends. A 1% increase in unemployment was related to an increase of 1.08 units in suicide attempt rate in men, and to a non-significant rise in women. Unemployment accounted for 48.3% of the total 8492 suicide attempt cases in the five initial years of the downturn (2008–2012).
Economou, M., Madianos, M., Peppou, L.E., Theleritis, C., et al. (2013)	Greece	Time Trend Analysis	2008 GFC	MDD (SCID-I), SI, & suicide attempt	An increase in the prevalence of SI was observed in men (7.1% vs. 4.4%), but not in women. The prevalence of SI increased among respondents aged 55–64 y.o. (7.2% vs. 1.9%), while it decreased in those <24 years (4.9% vs. 13.9%). The significant predictors of SI in 2011 were the presence of MDD, financial hardship, a history of suicide attempt, and low interpersonal trust. An increase in the prevalence of suicide attempt was observed in men (2.0% vs. 0.4%), but not in women. Odds of suicide attempt were more likely for people who fulfilled DSM-IV criteria for MDD (OR 97.39), men vs. women (OR 12.26), married vs. unmarried (OR 53.29), and a history of suicide attempt (OR 14.41).
Madianos, M., Economou, M., Alexiou, T., et al. (2010)	Greece	Time Trend Analysis	2008 GFC	MDD (SCID-I), SI, & suicide attempt	The 1-month prevalence rates of MDD in 2008 among males and females were 2.4% and 3.8%, respectively, compared to 4.6% in males and 8.8% in females in 2009. Unemployment increased odds of MDD 1.65× in 2008 and 1.28 times in 2009. Personal economic distress increased the probability (1.33×) of developing MDD by 2009. Among economically distressed respondents, SI was found in 35.0% in 2008 and 48.6% in 2009. In 2008, 0.6% of the sample reported that they had recently attempted suicide compared to 1.1% in 2009.
Hong, J., Knapp, M., McGuire, A. (2011)	South Korea	Time Trend Analysis	Late-1990’s Asian Financial Crisis	Depression, SI, & suicide attempt	The lowest income groups have the highest risk for depression, SI, or suicide attempt and SI and suicide attempt exhibited clearer income-gradient curves in more recent years. For depression, inequality increased sharply 1998–2001, and remained relatively stable thereafter. Inequality in the prevalence of SI increased gradually over time. In the case of suicide attempts, inequality decreased 1998–2001, but increased dramatically 2005–2007.
Economou, M., Peppou, L.E., Souliotis, K., et al. (2019)	Greece	Cross-sectional Study	2008 GFC	MDD (SCID) & SI	Being in the highest income category was protective against MDD (OR 0.37), while financial difficulties were a predictor of MDD (men OR 1.23; women OR 1.12). For men income increased the prevalence of suicidality (low OR 0.35; middle OR 0.22; high OR 0.19), but this was not significant for women. Financial difficulties were not significantly associated with suicidality once adjusted for income, MDD, education and employment.
Konstantakopoulos, G., Pikouli, K., Ploumpidis, D., et al. (2019)	Greece	Cross-sectional Study	2008 GFC	Diagnosis & suicide attempt	The rate of unemployment among new cases increased from 9.65% in 2008 to 26.17% in 2013. Compared to 2008, in 2013 patients with the following diagnoses were more likely to be unemployed: Anxiety (OR 7.49), and depressive disorders (OR 4.58). The odds of an individual referred after a suicide attempt being unemployed increased 19.29× in 2011 compared to 2008.
Ntountoulaki, E., Paika, V., Papaioannou, D., et al. (2017)	Greece	Cross-sectional Study	2008 GFC	Mental diagnoses (MINI) and suicide risk (RASS)	Female sex, lifetime history of mental disorders, and the perceived impact of the crisis were the variables most strongly associated with risk. The relationship between the perceived impact of the crisis and suicidal risk was significantly greater for those diagnosed with any mental disorder, and specifically, for those diagnosed with MDD (b = 0.091) and GAD (b = 0.092) compared to those without.
Gassman-Pines, A., Oltmans Ananat, E., Gibson-Davis, CM. (2014)	USA	Cross-sectional Study	2008 GFC	SI, plans, & attempts	Job loss among 1% of the working-age population increased the probability of adolescent girls reporting SI by 2.0% and suicide plans by 2.2%. Predicted probabilities indicated that job losses among 1% of the working-age population increased non-Hispanic Black adolescents’ SI by 2.3%, suicide plans by 3.1%, and suicide attempts by 2.0%. Job loss did not affect suicide-related behaviors among boys, non-Hispanic Whites, or Hispanics.
Miret, M., Caballero, F.F., Huerta-Ramirez, R., et al. (2014)	Spain	Cross-sectional Study	2008 GFC	SI, plans & attempts (WHO CIDI 3.0)	The factor most highly associated with lifetime SI or attempts in all age groups was having depression or anxiety. In the younger group, being unemployed or having work disability was associated with SI. In those aged 65 and older, a trend was observed to associate the presence of household financial problems with higher SI.
Vanderoost, F., van der Wielen, S., van Nunen, K., et al. (2013)	Belgium	Cross-sectional Study	2008 GFC	SI & depressive sx	An increase in SI was associated with the following characteristics: Being single, having difficulty making ends meet, having unsatisfying social contact, having poor self-rated health, having lost one’s employment in the past year, having had to cope with job insecurity, and having depressive complaints. No statistically significant association was found between suicidal thoughts and age, sex, education, or confrontation with employment loss of someone close.

Abbreviations in Table 3: Beck Hopelessness Scale (BHS); Composite International Diagnostic Interview (CIDI); Diagnostic and Statistics Manual (DSM); Generalized Anxiety Disorder (GAD); Global Financial Crisis (GFC); incident rate ratio (IRR); Major Depressive Disorder (MDD); Suicidal Scale of the Mini-International Neuropsychiatric Interview (MINI); odds ratio (OR); Richmond Agitation Sedation Scale (RASS); Structured Clinical Interview for DSM-IV Axis I Disorders (SCID-I); suicidal ideation (SI); symptom(s) (sx); versus (vs); United Kingdom (UK); United States of America (USA); World Health Organization (WHO); years old (y.o.).

**Table 4:** Summary of included articles on suicide (N = 46).

Author & Date	Country of Study	Study Type	Recession Studied	Mental Health Outcomes	Key Findings
Tanji, F., Kakizaki, M., Sugawara, Y., et al. (2015)	Japan	Prospective Cohort Study	Late-1990’s Asian Financial Crisis	Suicide	SMR increased from pre-1998 to post-1998 from 4.6 to 27.8 per 100,000 person-years. After 1998, neuroticism became a significant factor associated with increased risk (HR 2.45).
Rojas, Y., Stenberg, SA. (2016)	Sweden	Retrospective Cohort Study	2008 GFC	Suicide	Eviction was significantly related to suicide, with a corrected OR of 9.21. When substance abuse (OR = 4.82), mood disorders (OR = 4.94), and schizophrenia (OR = 7.36) are included in the analysis, the effect of eviction remains significant but decreases considerably in strength (OR 5.94).
Padmanathan, P., Bould, H., Winstone, L., et al. (2020)	High income countries, populations >20 million	Time Trend Analysis	2008 GFC	Suicide	An upturn in youth suicide predated the economic recession in the UK, USA, and Canada. Both the UK and USA experienced an acceleration in the rise in suicide rates after the economic recession. No statistical evidence that countries with rising suicide rates were more likely to have been affected by the 2008 recession. Countries with rising suicide rates had higher levels of income inequality and GDP in 2008.
Soleymani, M., Yip, P.S.F. (2020)	South Korea	Time Trend Analysis	Late-1990’s Asian Financial Crisis, 2008 GFC	Suicide	Increase in ratio of suicide rates between 1997 from 1996 of 1.94 for females and 1.98 for males. This remained persistently high following the 1997 economic downturn in South Korea leading into the 2008 economic recession. In 2008 vs. 2007, the ratio of suicide rates was 1.01 for females and 1.03 for males but was observed to have a possible six-month lag after the downturn.
Demirci, S., Konca, M., Yetim, B., et al. (2020)	USA	Time Trend Analysis	2008 GFC	Suicide	The 2008 crisis was found to explain 30% of the change in US suicide rates in both the short-term and long-term up to the end of the data set in 2016. Unemployment rates were found to have no effect on suicides in the short and long run in the US.
Ibrahim, S., Hunt, I.M., Rahman, M.S., et al. (2019)	UK	Time Trend Analysis	2008 GFC	Suicide	Compared to pre-recession, among those who died in the recession period men were more likely to be aged 45–54 (OR 1.26), women were more likely to be single (OR 1.27), and both genders were more likely to be unemployed (men OR 1.15; women OR 1.26). Suicide deaths associated with affective disorders did not change significantly between any period.
Alexopoulos, E.C., Kavalidou, K., Messolora, F. (2019)	Greece	Time Trend Analysis	2008 GFC	Suicide	Males in armed forces, agriculture, and elementary occupations had an elevated risk of suicide overall (CMR 1.56), a significant decline in their ratios was evident between 2010 and 2013. Working-age females were affected in various occupational groups: Technologists and associate professionals (CMR 8.66), agricultural and fishery workers (CMR 4.77), machine operators and assemblers (CMR 28.57), especially in the age group of 50–59 y.o.
Lopez-Contreras, N., Rodriguez-Sanz, M., Novoa, A., et al. (2019)	Spain	Time Trend Analysis	2008 GFC	Suicide	Pre-crisis the adjusted SMR was higher among men with lower level of education, however this decreased during the crisis periods. Individual unemployment was not correlated with suicide rate in either sex; however, suicide risk increased in men living in neighbourhoods with higher unemployment in the late crisis period while at the same time decreasing risk in women in these areas.
Yoon, J.H., Jung, S.J., Choi, J., et al. (2019)	South Korea	Time Trend Analysis	Late-1990’s Asian Financial Crisis, 2008 GFC	Suicide	Three major peaks in 1998 (1997–1998 Asian financial crisis), 2003 (revenue generating measures led to 2× increase in credit card defaulters), and 2008–2010 (2008 GFC). During the first and second periods, the rise was prominent among lower socio-economic occupation groups (unskilled manual and service-trade). Since 2008 there has been a steady rise in suicide rate among older male officers and managers, while suicide rate among females has continued to decline after a peak in 2009. The most vulnerable groups were middle-aged service and trade workers when the GDP growth rate declined, and middle-aged skilled manual workers when the unemployment rate aggravated.
Basta, M., Vgontzas, A., Kastanaki, A., et al. (2018)	Greece	Time Trend Analysis	2008 GFC	Suicide	Men <40 y.o. had the highest unemployment rates during the economic crisis (45%) and the lowest SMR (4.1), whereas men 40–64 y.o. had lower unemployment (20%) but the highest SMR (28.42). Women <40 y.o. had the highest unemployment (53%) and lowest SMR (0 cases), while middle-aged women (40–64 y.o.) had lower unemployment (27%) and higher SMR (4.8).
Alvarez-Galvez, J., Salinas-Perez, J.A., Rodero-Cosano, M.L., et al. (2017)	Spain	Time Trend Analysis	2008 GFC	Suicide	The overall suicide rate remained relatively stable from 2007 to 2011, but there was an increase for men post 2008–2011 and 2011–2014. During the second period of recession (2011–2014), the rate of suicide increased between 0.003 and 0.004 every month. Both unemployment and per capita GDP were positively related to suicide trends, while social expenditure did not show a statistically significant association.
Ruiz-Perez, I., Rodriguez-Barranco, M., Rojas-Garcia, A., et al. (2017)	Spain	Time Trend Analysis	2008 GFC	Suicide	Suicide rate decreased overall 2002–2012, but the downward trend reversed in 2008–2009 and 2012. Rates increased 7% in 2008 and 6% in 2009 compared to 2007, affecting men from all age groups except the oldest. For men 50–64 the suicide rate increased 17% and 18% during 2008 and 2009, respectively. For women, suicide rates increased 2007–2008 then decreased (17% lower in 2010 vs. 2007) before peaking again to 3% below 2007 level in 2012.
Borrell, C., Mari-Dell’Olmo, M., Gotsens, M., et al. (2017)	Spain	Time Trend Analysis	2008 GFC	Suicide	Total SMR were higher among men than women. For men, an inverse relationship with educational level was observed in all three time periods. Among women, the rates of suicide were lower and did not show such a clear relationship.
Agrrawal, P., Waggle, D., Sandweiss, DH. (2017)	USA	Time Trend Analysis	2008 GFC	Suicide & Murder-Suicide	SMR 2005–2007 averaged 11.21 per 100,000, but for 2008–2013 the average climbed to 12.53 per 100,000. Suicides from a ‘financial problem’ rose from 11.55% to 12.41% of instances from 2005–2007 vs. 2008–2013. Murder-suicides known to be triggered by a ‘financial problem’ peaked at 10.98% in 2009 (average rate 2005–2013 = 7.56%). Suicide rates are observed to peak about two years from the low point in the capital markets. 74.18% of the variation in annual SMR was explained by market risk premium, unemployment rate, and real GDP growth rate.
Houle, J.N., Light MT. (2017)	USA	Time Trend Analysis	2008 GFC	Suicide	1% increase in the foreclosure rate was found to add 1.12 additional suicides per 100,000. Relationship was attenuated by recessionary period factors (falls to 0.23 additional suicides per 100,000). Foreclosure effects were statistically significant for white men only = 0.91 (out of 6 racial-sex models).
Jeon, S.Y., Reither, E.N., Masters, R.K. (2016)	South Korea, Japan	Time Trend Analysis	Late-1990’s Asian Financial Crisis, 2008 GFC	Suicide	In Japan, suicide rates spiked in 1995–2000, then stabilized at about 26 per 100,000 PYL out to 2010. In South Korea, suicide rates increased sharply after 2000 reaching 35.9 in 2010. SMR was higher among men than women in each period. The spike seen in Japan in the late 1990s was caused almost entirely by men, whereas trends in South Korea were similar between men and women throughout the study period (1985–2010).
de Beurs, D.P., Hooiveld, M., Kerkhof, A.J.F.M., et al. (2016)	The Netherlands	Time Trend Analysis	2008 GFC	Suicide & suicide attempts	From 2007 to 2013, there was a sudden significant increase in the suicide rate (b = 0.32). Compared to pre-2007, the peak age of male suicides shifted from 30–39 y.o. to 60–69 y.o. in 2008–2013, and the median age peak of female suicides shifted from 30–39 y.o. to 50–59 y.o. The median age of suicide attempt for males rose from 30–39 y.o. to 40–49 y.o. For female suicide attempts, the median age changed from 20–29 to 50–59. History of MDD of male patients who died by suicide decreased from 60% to 46% and for females, it decreased from 47% to 40%.
Merzagora, I., Mugellini, G., Amadasi, A., et al. (2016)	Italy	Time Trend Analysis	2008 GFC	Suicide	No significant difference in the suicide rate during the economic crisis; however, the lowest annual rate was registered in 2007 and the highest in 2009 and 2013. There was no significant relationship between employment status or health status and suicide risk during the economic crisis. The likelihood of suicide during the downturn compared to the pre-crisis period was 1.6 times lower for those who were married or cohabitating vs. divorced, widowed, or single, and 1.5 times less for people 25–34 y.o. vs. other age categories.
Milner, A.J., Niven, H., LaMontagne, A.D. (2015)	Australia	Time Trend Analysis	2008 GFC	Suicide	Compared to managers, RR of suicide increased from 2001–2006 among technical and trade workers (RR 1.87 to 4.25 in 2008, 5.20 in 2010), community service workers (RR 1.45 to 3.85 in 2007, 4.17 in 2010). For females, RR of suicide increased compared to managers among technical and trade workers (RR 4.33 in 2007, and 4.01 in 2008) and machinery workers (RR 1.67 in 2001–2006 to 4.90 in 2009).
Harper, S., Charters, T.J., Strumpf, E.C., et al. (2015)	USA	Time Trend Analysis	2008 GFC	Suicide	During the GFC, a 10-unit decrease in the ICEI was associated with an increase of 0.14 suicide deaths per 100,000 population, or a roughly 1% increase. A 10-unit decrease in the ICEI increased SMR by 0.28 deaths per 100,000 population among men but had no effect on women. Effects were generally similar by race but differed by age group driven by a protective effect among those aged 65 and over.
Rachiotis, G., Stuckler, D., McKee, M., et al. (2015)	Greece	Time Trend Analysis	2008 GFC & Austerity Period	Suicide	The SMR after 2010, when austerity began, was significantly higher than in the period 2003–2010 (4.42 vs. 3.35/100,000 population)—for men from 5.75 to 7.43, respective and among women from 1.17 to 1.55, respectively. For working men aged 20–59, the rate increased from 6.58 to 8.81; and among women ages 20–59 there was an increase from 1.37 to 1.84. There was no association between suicides in working aged men or women and GDP. Each 1% rise of unemployment rates in men 20–59 y.o. was associated with a 0.19/100,000 population rise in suicides. Unemployment appeared to mediate the association with the austerity period.
Fowler, K.A., Gladden, M., Vagi, K.J., et al. (2015)	USA	Time Trend Analysis	2008 GFC	Suicide	Suicides with eviction or foreclosure circumstances doubled from 2005 to 2010 and accounted for 1–2% of all suicides with a peak in 2009. Most suicides occurred before the actual loss of housing (79%). 37% of cases experienced a crisis related to the eviction or foreclosure (e.g., a court hearing, an eviction notice, the date on which the person was to vacate the dwelling) within 2 weeks of the suicide.
Corcoran, P., Griffin, E., Arensman, E., et al. (2015)	Ireland	Time Trend Analysis	2008 GFC	Suicide & self-harm	By the end of 2012, the estimated male suicide rate was 23.8 per 100,000; 8.7 per 100,000 or 57% higher than if the pre-recession trend had continued; an excess of 476 male suicide deaths occurred from 2008–2012. The estimated female suicide rate was like what would have been observed if the pre-recession trend had continued or if it had leveled off. Male self-harm was 74.1 per 100,000 or 31% higher than the pre-recession trend, whereas female self-harm was 63.2 per 100,000 or 22% higher. The associated excess of hospital-treated self-harm in 2008–2012 was 5029 male and 3833 female presentations.
Norstrom, T., Gronqvist, H. (2015)	27 European Countries, Canada, Australia, and USA	Time Trend Analysis	2008 GFC	Suicide	Unemployment increased after 2007 in all country groups except the Bismarckian. The male SMR increased in the eastern European countries, but little or not at all in other country groups. The female SMR was generally stable or decreased. The estimated effect was strongest in the two country groups with the weakest unemployment protection (eastern and southern Europe), while the weakest effect was found in those with the strongest protection (Bismarckian and Scandinavian countries).
Saurina, C., Marzo, M., Saez, M. (2015)	Spain	Time Trend Analysis	2008 GFC	Suicide	An increase was observed, which was much greater in women, in the relative risks of suicide from 2009 onwards. This increase was only found in municipalities with 10,000 or more inhabitants and was only statistically significant for working-aged women (16–65 y.o.) In no case was the unemployment rate to found to be significantly associated with the suicide rate.
Fountoulakis, K.N., Kawohl, W., Theodorakis, P.N., et al. (2014)	29 European countries	Time Trend Analysis	2008 GFC	Suicide	Total SMR and male SMR were related to all economic variables except GDP per capita. Female SMR was only related to unemployment. Except for 4 countries (Bulgaria, Estonia, Finland, and Switzerland), for the other 25 countries a halt in decreasing SMR was seen during 2006–2008, followed by an increase, which in half of them was temporary.
Phillips, J.A., Nugent, C.N. (2014)	USA	Time Trend Analysis	2008 GFC	Suicide	A 1% increase in unemployment is associated with an increase of 1.6% in SMR. Rising unemployment had a more adverse effect on SMR in states with relatively high mean levels of female labour force participation, where in states with >55.7% female labour force participation, a 5% increase in the unemployment rate was associated with a 10% increase in total SMR.
Coope, C., Gunnell, D., Hollingworth, W., et al. (2014)	England, Wales	Time Trend Analysis	2008 GFC	Suicide	Increases in SMR in 2008 were seen in 16–24 (26% rise) and 35–44 y.o. men (14% rise). In women peak SMR in 16–24, 25–34, and 35–44 y.o. in 2008 with 28%, 20% and 47% rises compared to 2006, and for 45–54 y.o. in 2010. These changes in trends in SMR in 16–34 y.o. coincide with increases in financial strain. There was no consistent pattern of change across income groups.
Houle, J.N., Light, MT. (2014)	USA	Time Trend Analysis	2008 GFC	Suicide	The within-state SMR increased by 0.10 for every 1% increase in the foreclosure rate from 2005 to 2010. A large proportion of the foreclosure effect is explained by state-level structural disadvantage. Within-state foreclosure effect was significant for those 30–45 y.o. and 46–64 y.o., and about twice as large for those nearing retirement compared with those 30–45 y.o. Real estate-owned foreclosures alone explained 18% of the within-state variance in suicides among those 46 to 64 y.o.
Pompili, M., Vichi, M., Innamorati, M., et al. (2014)	Italy	Time Trend Analysis	2008 GFC	Suicide	When the year 2006 was set as a reference point, suicide rates in men 25–64 y.o. were stable in 2007 and then increased progressively over the next few years, with a 12% increase between 2006 and 2010. In contrast, in the younger and older male age groups, and in females, the suicide rates did not increase significantly during the 5 years investigated.
Milner, A., Morrell, S., LaMontagne, AD. (2014)	Australia	Time Trend Analysis	2008 GFC	Suicide	The difference in suicide rate ratio (SRR) from 2006 to 2008 for economically inactive males (SRR 1.22) and for females rose in 2007 and 2008 (SRR 1.12; RR 1.19, respectively). Unemployed males had a SRR of 10.64 compared with those employed and unemployed females had a SRR of 5.47 compared with employed females.
Fountoulakis, K.N., Gonda, X., Dome, P., et al. (2014)	Hungary	Time Trend Analysis	2008 GFC	Suicide	Suicide rates were dropping steadily until 2006. In 2007–2011, rates were stable with a tendency to increase. The correlation between the general population SMR and the unemployment population SMR was strong (0.91). The correlations between the unemployment rate and suicide rates were strongly negative both for the general and for the unemployed populations (−0.65 and −0.55, respectively).
Lopez Bernal, J.A., Gasparrini, A., Artundo, C.M., et al. (2013)	Spain	Time Trend Analysis	2008 GFC	Suicide	There was an 8% step increase in the suicide rate associated with the financial crisis. Although the stratified analyses suggest a greater increase in the Mediterranean and Northern areas, in males and in younger age groups, the low statistical power when testing for interaction prevent any firm conclusions being made on a differential effect by sub-groups.
Fountoulakis, K.N., Savopoulos, C., Siamouli, M., et al. (2013)	Greece	Time Trend Analysis	2008 GFC	Suicide	No gross change in suicidality was seen in Greece during the 2008 economic crisis in this study. The correlations between suicidal rates and unemployment and growth rate were essentially zero (0.04 and −0.08, respectively).
Garcy, A.M., Vagero, D. (2013)	Sweden	Time Trend Analysis	2008 GFC	Suicide	During the recession there was no excess hazard of mortality from suicide or events of undetermined intent (hazard ratio [HR] = 1.29). Post-recession, there was an excess hazard of suicide for unemployed men (HR = 1.43) but not unemployed women. However, for unemployed women with no health history there was a modest hazard of suicide.
Saurina, C., Bragulat, B., Saez, M., et al. (2013)	England	Time Trend Analysis	2008 GFC	Suicide	Compared with 1993–2007, from 2008 to 2010, there were statistically significant variations only in suicide rates, not in the number of suicides, and only at the regional level, not at the level of England as a whole. For men, statistically significant unemployment rates were positively associated with age-standardised suicide rates in southwest, northwest and northeast England.
Chang, S.S., Stuckler, D., Yip, P., et al. (2013)	54 Countries	Time Trend Analysis	2008 GFC	Suicide	The overall male SMR rose 3.3%, with an excess of 5124 suicides in 2009. These were mainly seen in 27 European countries (4.2%; 2937) and 18 American countries (6.4%; 3175). There was also a small rise in suicide rates in women in American countries (2.3%), but no increase among European women. In countries with low unemployment pre-crisis < 6.2% the correlation for men was 0.48 and in countries with high unemployment (≥6.2%) it was insignificant.
Barr, B., Taylor-Robinson, D., Scott-Samuel, A., et al. (2012)	England	Time Trend Analysis	2008 GFC	Suicide	Between 2008 and 2010, there were 846 more suicides among men than would have been expected based on historical trends, and 155 among women. Each 10% increase in the number of unemployed men was significantly associated with a 1.4% increase in male suicides. About 2/5 suicides among men during the recession were attributed to rising unemployment.
Nandi, A., Prescott, M.R., Cerda, M., et al. (2012)	USA	Time Trend Analysis	2008 GFC	Suicide	There was a negative association between rates of suicide and the ICEI at values greater than 105, indicating that rates of suicide in NYC were lowest when economic activity was greatest. The rate of suicide declined monotonically among whites, men, and adults <45 y.o. as the ICEI increased.
Luo, F., Florence, C.S., Quispe-Agnoli, M., et al. (2011)	USA	Time Trend Analysis	The Great Depression (1929–1933), 1937–1938, 1973–1975 Oil Crisis & 1980’s Recession	Suicide	The largest increase in SMR occurred during the Great Depression (1929–1933), when it surged from 18.0 in 1928 to 22.1 (the all-time high) in 1932. This increase of 22.8% was the highest recorded for any 4-year interval during the study period. The overall suicide rate also rose during 3 other severe recessions: The end of the New Deal (1937–1938), the oil crisis (1973–1975), and the double-dip recession (1980–1982). Not only did the overall suicide rate generally rise during recessions; it also mostly fell during expansions.
Vanthomme, K., Gadeyene, S. (2019)	Belgium	Cross-sectional Study	2008 GFC	Suicide	Unemployment is associated with an increased mortality rate difference of 3.4 deaths per 100,000 among men and 0.7 deaths per 100,000 among women for mortality due to “mental disorder”, excluding alcohol-related deaths.
Beautrais, A.L. (2018)	New Zealand	Cross-sectional Study	2008 GFC & Global Dairy Crisis (2015–2016)	Suicide	The single most common precipitant was existing mental illness in 28.6% of cases. All but 1 person was employed. Other precipitating factors were physical health problems (12.4%), conflict or arguments (8.1%), legal charges/issues (5.4%), financial problems (4.8%), acute alcohol or drug intoxication (3.2%), ‘life struggles’ (or accumulated problems; 2.1%), work problems (1.1%), recent death of a family member or friend (1.1%).
Kerr, W.C., Kaplan, M.S., Huguet, N., et al. (2017)	USA	Cross-sectional Study	2008 GFC	Suicide	Suicide rates were most strongly associated with county-level poverty rates for both men (14.128) and women (8.884). For women, foreclosure rates were negatively related to suicide rates (−1.447), whereas no relationship was found for men. Results by age group for foreclosure rates found a significant and positive relationship in those 45–64 y.o. (1.204), and a significant negative effect on suicide rates was found among those 65 y.o. and older (−7.388).
Coope, C., Donovan, J., Wilson, C., et al. (2015)	England	Cross-sectional Study	2008 GFC	Suicide	Compared to non-recession related suicides, people whose suicide was recession-related were older, had no history of psychiatric illness (39% vs. 24%), had not previously self-harmed (32% vs. 51%), did not seek mental health supports (21% vs. 29%), had not visited their GP (36% vs. 45%), had financial problems (76% vs. 23%), and financial responsibility for others (55% vs. 23%). Of those who had experienced financial difficulties, only 31% were unemployed. Eleven deaths (4%) were thought to be entirely due to financial/employment-related difficulties.
Hempstead, K.A., Phillips, JA. (2015)	USA	Cross-sectional Study	2008 GFC	Suicide	Personal circumstances, such as mental health problems, were cited in 81% of all suicide incidents. Interpersonal circumstances were more common among men than women (42% vs. 37%). External circumstances were recorded in 39.1% of suicides among men vs. 22.8% among women. External circumstances were the least common of the three categories but increased from 29.8% of suicide deaths in 2005 to 32.7% in 2010.
Reeves, A., McKee, M., Gunnell, D., et al. (2015)	24 European Countries	Cross-sectional Study	2008 GFC	Suicide	Each 1% rise in the male unemployment rate is associated with a 0.94% rise in male suicide rates. Unemployment–suicide associations were concentrated in working-age men: Each 1% rise in unemployment among those aged 25–64 y.o. was associated with a 1.39% rise in male suicide rates. Each 10 US dollar increase in government spending on active labour market programmes reduced the effect of a 1% rise in male unemployment on the male suicide rate by 0.026%, and a 1% rise in population with trust in others, reduced the association by 0.048%.

Abbreviations in Table 4: Comparative mortality rate (CMR); gross domestic product (GDP); Global Financial Crisis (GFC); Index of Coincident Economic Indicators (ICEI); Major Depressive Disorder (MDD); odds ratio (OR); person-years lived (PYL); relative risk (RR); suicide mortality rate (SMR); suicide rate ratio (SRR); United Kingdom (UK); United States of America (USA); versus (vs); years old (y.o.).

## Data Availability

No new data were created or analyzed in this study. Data sharing is not applicable to this article.

## Supplementary Materials

The following are available online at https://www.mdpi.com/article/10.3390/bs11090119/s1, Table S1: Extended summary of included articles, and Figure S1: Literature search flowchart.
