# Supplementary figures and images for: The Impact of Economic Recessions on Depression, Anxiety, and Trauma-Related Disorders and Illness Outcomes—A Scoping Review
Source: Behav Sci (Basel). 2021 Aug 31;11(9):119. doi: 10.3390/bs11090119 (PMC8464685; doi:10.3390/bs11090119)

Figure S1. Literature Search Flowchart

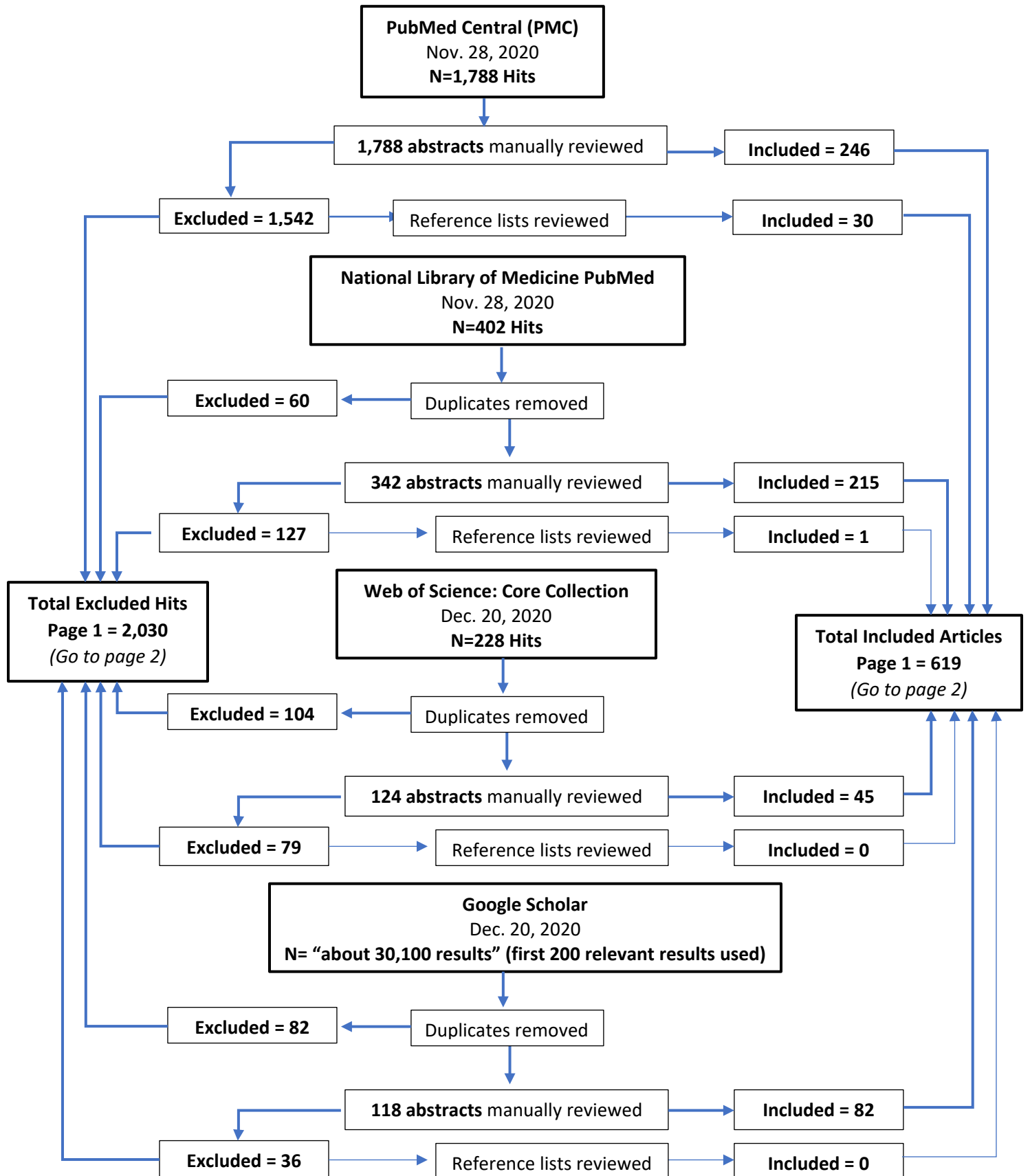

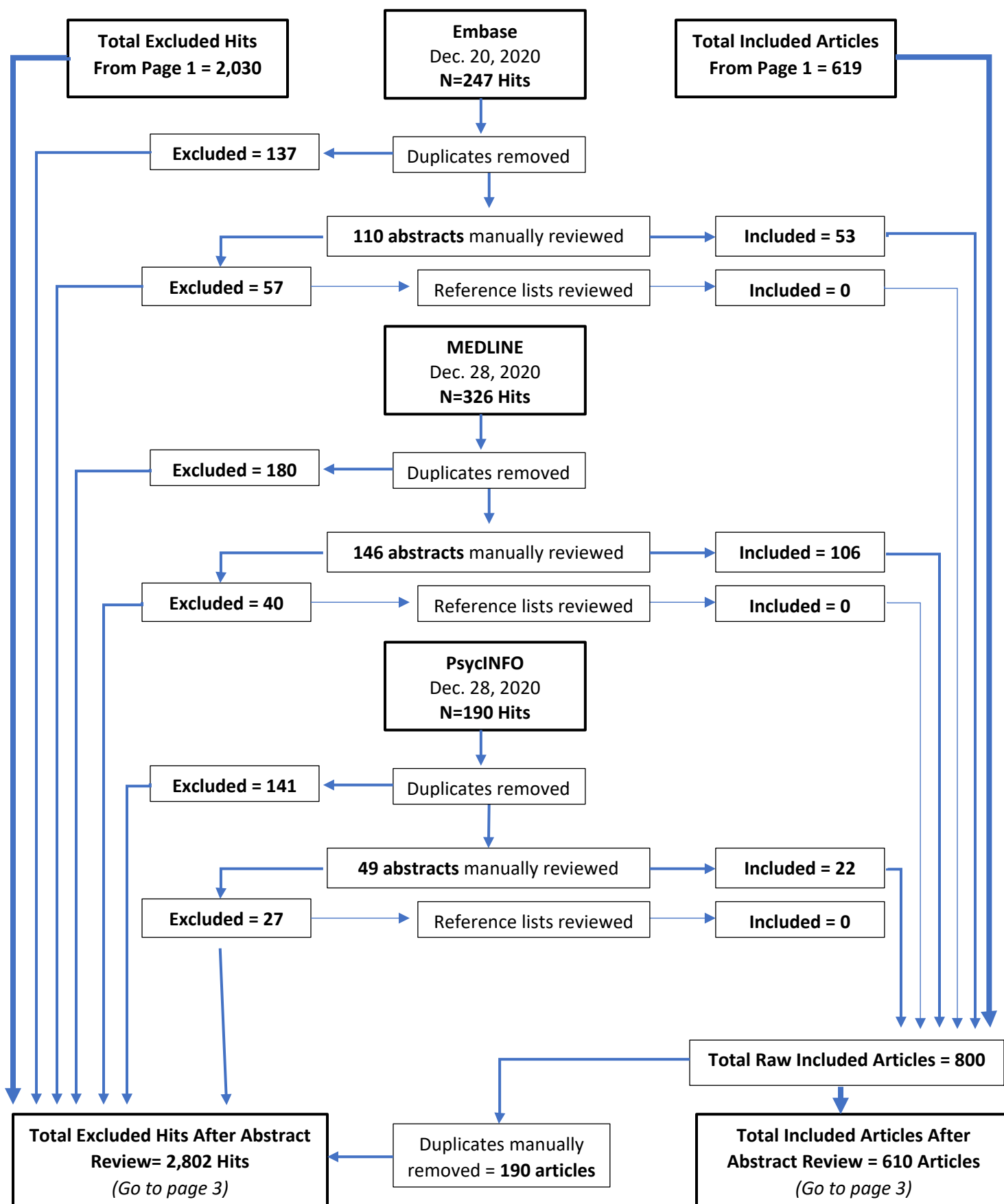

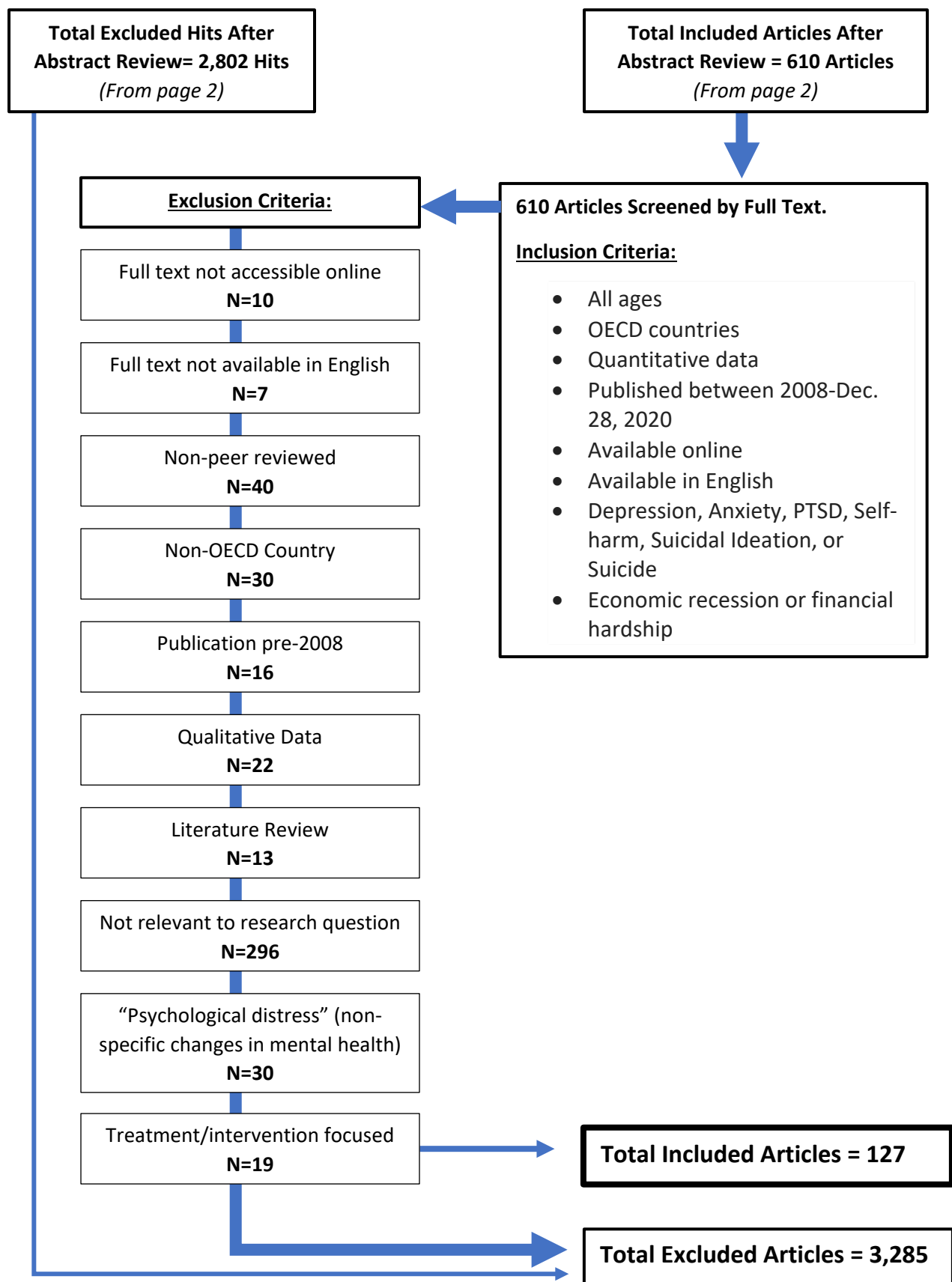

Supplement: Supplementary file 1 [file behavsci-11-00119-s001.zip › Figure S1. Literature Search Flowchart.pdf]
